# Pan-Cancer Analysis of Immune Cell Infiltration Identifies a Prognostic Immune-Cell Characteristic Score (ICCS) in Lung Adenocarcinoma

**DOI:** 10.3389/fimmu.2020.01218

**Published:** 2020-06-30

**Authors:** Shuguang Zuo, Min Wei, Shiqun Wang, Jie Dong, Jiwu Wei

**Affiliations:** ^1^Jiangsu Key Laboratory of Molecular Medicine, Medical School of Nanjing University, Nanjing, China; ^2^Nanjing University Hightech Institute at Suzhou, Suzhou, China

**Keywords:** immune cell infiltration, tumor microenvironment, lung adenocarcinoma, single-sample Gene Set Enrichment Analysis, prognosis

## Abstract

**Background:** The tumor microenvironment (TME) consists of heterogeneous cell populations, including malignant cells and nonmalignant cells that support tumor proliferation, invasion, and metastasis through extensive cross talk. The intra-tumor immune landscape is a critical factor influencing patient survival and response to immunotherapy.

**Methods:** Gene expression data were downloaded from The Cancer Genome Atlas (TCGA) and Gene Expression Omnibus databases. Immune cell infiltration was determined by single-sample Gene Set Enrichment Analysis (ssGSEA) depending on the integrated immune gene sets from published studies. Univariate analysis was used to determine the prognostic value of the infiltrated immune cells. Least absolute shrinkage and selection operator (LASSO) regression was performed to screen for the most survival-relevant immune cells. An immune-cell characteristic score (ICCS) model was constructed by using multivariate Cox regression analysis.

**Results:** The immune cell infiltration patterns across 32 cancer types were identified, and patients in the high immune cell infiltration cluster had worse overall survival (OS) but better progression-free interval (PFI) compared to the low immune cell infiltration cluster. However, immune cell infiltration showed inconsistent prognostic value depending on the cancer type. High immune cell infiltration (High CI) indicated a worse prognosis in brain lower grade glioma (LGG), glioblastoma multiforme (GBM), and uveal melanoma (UVM), and favorable prognosis in adrenocortical carcinoma (ACC), cervical squamous cell carcinoma and endocervical adenocarcinoma (CESC), cholangiocarcinoma (CHOL), head and neck squamous cell carcinoma (HNSC), liver hepatocellular carcinoma (LIHC), lung adenocarcinoma (LUAD), sarcoma (SARC), and skin cutaneous melanoma (SKCM). LUAD prognosis was significantly influenced by the infiltration of 13 immune cell types, with high infiltration of all but Type 2 T helper (Th2) cells correlating with a favorable prognosis. The ICCS model based on six most survival-relevant immune cell populations was generated that classified patients into low- and high-ICCS groups with good and poor prognoses, respectively. The multivariate and stratified analyses further revealed that the ICCS was an independent prognostic factor for LUAD.

**Conclusions:** The infiltration of immune cells in 32 cancer types was quantified, and considerable heterogeneity was observed in the prognostic relevance of these cells in different cancer types. An ICCS model was constructed for LUAD with competent prognostic performance, which can further deepen our understanding of the TME of LUAD and can have implications for immunotherapy.

## Introduction

Cancer is a highly heterogeneous disease involving complex interactions between the malignant cells and the tumor microenvironment (TME). The latter consists of various immune cells, mesenchymal-origin cells, and the extracellular matrix (ECM) (1, 2), which influence all stages of tumorigenesis by directly interacting with the tumor cells (3, 4). The immunological component of the TME acts as a two-edged sword that can either suppress or promote tumor development (5). The infiltrating immune cells in the TME are critical players affecting tumor growth and progression, as well as therapeutic outcomes and patient prognosis (6–8).

Lung cancer is the leading cause of cancer-related deaths worldwide, with 2,093,876 newly diagnosed cases and 1,761,007 deaths recorded in 2018 alone (9). Lung adenocarcinoma (LUAD) is the most common histological subtype (10). Studies show the infiltration of multiple immune cells in the lung TME (4, 11), including that of T lymphocytes, B cells, dendritic cells (DCs), macrophages, and natural killer (NK) cells (12). In fact, the relative proportion of these tumor-infiltrating immune cells creates the microenvironment of lung cancer (4). Therefore, it is not surprising that immunological parameters of LUAD, such as the infiltrating T cells, are important discriminants of tumor stratification, clinical outcomes, and patient survival (13, 14). The previous study has shown that the tumor-infiltrating immune cells are correlated with the development and progression of LUAD (11). The type and level of immune cells not only have a prognostic value but also affect the response of immunotherapy. However, there are few studies to analyze the correlation between tumor-infiltrating immune cells and the prognosis of patients with LUAD.

The recent advances in genomic sequencing and bioinformatics have enabled high throughput analysis and interpretation of complex disease-related datasets, which are ideal approaches to quantify the tumor-infiltrating immune cells of various cancers (15). Single-sample Gene Set Enrichment Analysis (ssGSEA) is an extension of Gene Set Enrichment Analysis (GSEA), which calculates separate enrichment scores for each pairing of a sample and gene set (16). In this manner, ssGSEA transforms a single sample's gene expression profile to a gene set enrichment profile. By defining immune cell-related gene sets, the enrichment score of the gene set can represent the density of tumor-infiltrating immune cells. This transformation allows researchers to characterize tumor-infiltrating immune cells in the TME rather than through immunohistochemistry and flow cytometry.

In the present study, we analyzed the immune cell infiltration pattern of a pan-cancer cohort that includes 32 cancer types using the ssGSEA method. Least absolute shrinkage and selection operator (LASSO) regression was used to screen for the most survival-relevant immune cells. Cox regression analysis was to establish an ICCS model in both training and validation cohorts of LUAD. We believe that the ICCS could assist in predicting the survival of LUAD patients and can further deepen our understanding of the TME of LUAD.

## Methods

### Data Sources

Gene expression data and corresponding clinical annotations of tumor samples were obtained from The Cancer Genome Atlas (TCGA; https://www.cancer.gov/tcga) and Gene Expression Omnibus (GEO; http://www.ncbi.nlm.nih.gov/geo/) databases. RNAseq data (RSEM gene-normalized) and clinical annotations for the TCGA cohorts (10,150 tumors across 32 cancer types) were obtained from the UCSC Xena browser (https://xenabrowser.net; Table S1). After removing normal tissue and non-primary tumor samples, 9,112 primary tumor samples were selected. The gene expression levels were analyzed using the Illumina HiSeq 2000 RNA Sequencing platform, and all Level-3 data were downloaded. Microarray and clinical data of LUAD patients were obtained from the GSE31210 (*n* = 226) (17, 18), GSE37745 (*n* = 106) (19, 20), and GSE50081 (*n* = 128) (21) datasets of the GEO database. All microarray data had been generated using the Affymetrix HG-U133 Plus 2.0 platform. The LUAD samples in the TCGA database were used as the training set and those from GEO datasets as the validation sets.

### Acquisition of the Immune Cell-Related Gene Sets

Gene sets specific for immune cell populations were obtained from the following studies: Bindea et al. (3), Zheng et al. (22), Charoentong et al. (23), Racle et al. (24), Tirosh et al. (25), and Angelova et al. (26). The expression data published by Zheng et al. (22) and Tirosh et al. (25) were generated using single-cell sequencing and measured in the other studies (3, 23, 24, 26) by microarray profiling.

### Single-Sample Gene Set Enrichment Analysis

The infiltration level of the different immune cell populations was determined by ssGSEA (27) in the R Bioconductor package Gene Set Variation Analysis (GSVA, v.3.5) using default parameters. The ssGSEA algorithm is a rank-based method that defines a score representing the degree of absolute enrichment of a particular gene set in each sample. The gene sets from the published studies were fed into the ssGSEA algorithm. Pearson's correlation coefficient was used to calculate the correlation of the ssGSEA scores across the gene sets (Figure S1). The ssGSEA scores for most immune cell populations obtained using the gene sets from Angelova et al. (26) were either highly correlated or mildly anti-correlated and therefore excluded. For the gene sets that were included in no less than two published studies (Table S2), those with ssGSEA scores consistent with known immune cell markers were retained (Figure S2), as were gene sets that were not duplicated across the different studies. Finally, a total of 46 gene sets (Table S3) representing distinct immune cell populations were selected, and the ssGSEA scores of each were calculated across 9,112 samples in the pan-cancer cohort. The correlation of the ssGSEA scores was calculated by Pearson's method.

### Unsupervised Clustering

An unsupervised *k*-means clustering method was used for patient classification based on the ssGSEA scores of infiltrated immune cells. An fpc package (v2.2-2, https://CRAN.R-project.org/package=fpc) was used to determine the optimal number of clusters, followed by identification of the cluster information through *k*-means function in the statsr package (v0.1-0, https://CRAN.R-project.org/package=statsr).

### Survival Analysis

Univariate and multivariate Cox proportional-hazards regression models were analyzed using the Survival package in R. Kaplan–Meier survival curves were plotted and compared using the log-rank test. The ggplot2 package was used for data visualization. The LASSO (28) was then used to screen variables that were highly correlated with survival outcomes, and those with a regression coefficient larger than zero were selected. Based on the multivariate Cox proportional hazards model, an ICCS model was generated as follows:

$$\begin{array}{l} ICCS=\overset{n}{\underset{i=1}{\sum }}S_{i}*\beta_{i} \end{array}$$

where *n* is the number of selected immune cells, S_i_ is the ssGSEA score of the immune cell population *i*, and β_*i*_ is the coefficient of *i*. Using the median ICCS as the cutoff value, the patients were divided into low- and high-ICCS groups. The receiver operating characteristic (ROC) curve was used to evaluate the accuracy of the ICCS model by comparing the area under the curves (AUCs). A *P* < 0.05 was considered statistically significant.

## Results

### The Reliability of the Identified Gene Sets for Single-Sample Gene Set Enrichment Analysis

**Figure 1:**
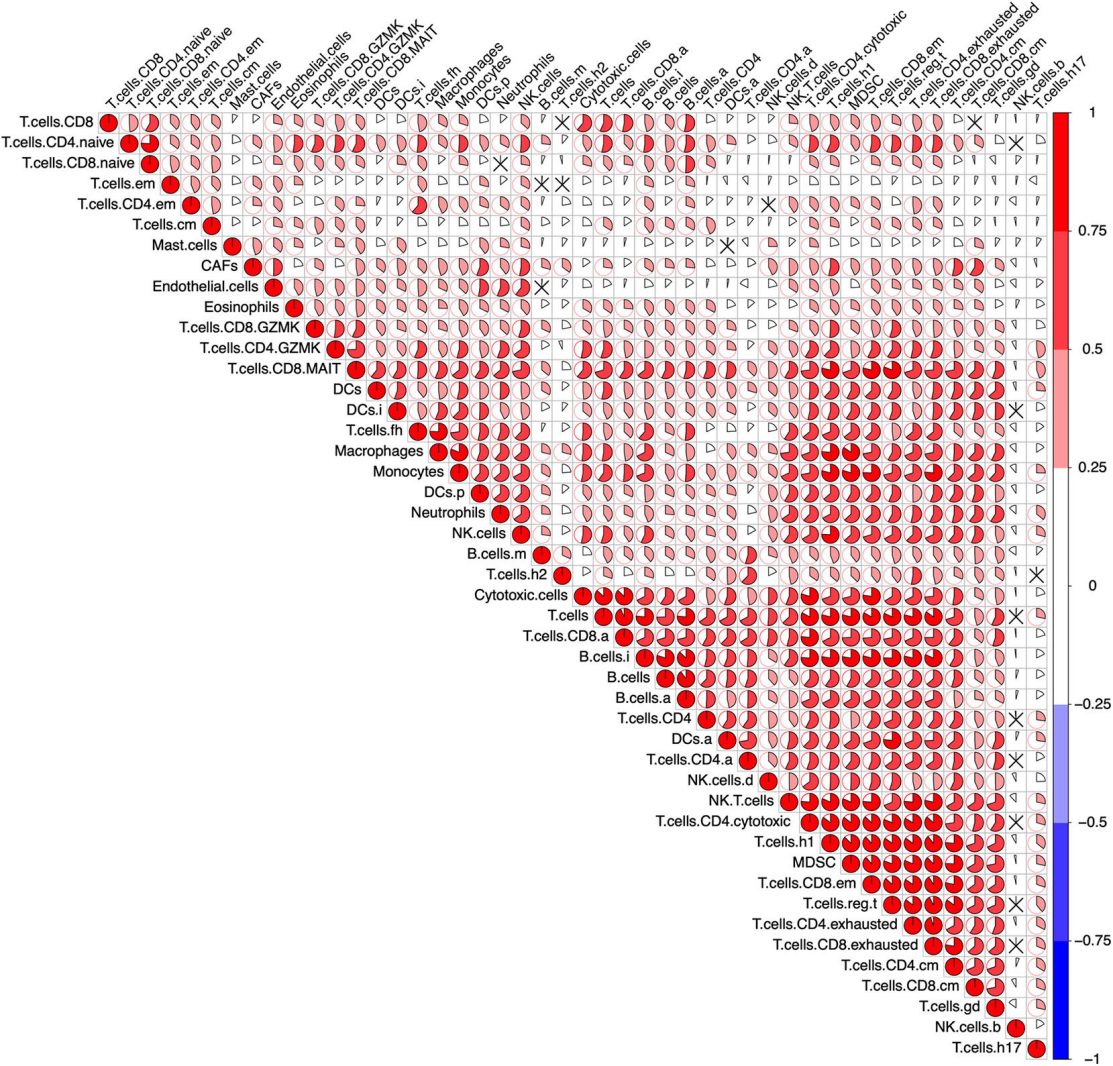
Correlation of the immune cells across the pan-cancer cohort. After calculating the single-sample Gene Set Enrichment Analysis (ssGSEA) score representing the immune cells in the pan-cancer cohort (9,112 patients), a Pearson's correlation analysis between each immune cell was performed. The result was visualized using the R package “corrplot.” The size of the sector area and the gradient of colors represented the correlation coefficient R. “ × ” means no statistical significance (*P* > 0.05).

To determine the reliability of the 46 selected gene sets (Table S3), a correlation analysis between the ssGSEA scores which represent each immune cell population was performed across all samples in the pan-cancer cohort. The ssGSEA scores of most immune cell populations exhibited a positive correlation without any anti-correlation, indicating the reliability of the gene sets (Figure 1). In addition, the active, immature, and mature B cells exhibited the highest mean ssGSEA scores in diffuse large B-cell lymphoma (DLBC), and most T cells showed the highest ssGSEA scores in thymoma (THYM) or DLBC. These results are consistent with most published data and again underscore the reliability of the gene sets representing distinct immune cell populations (Figure S3).

### Immune Cell Infiltration-Based Classification in Different Cancers Shows Prognostic Heterogeneity

**Figure 2:**
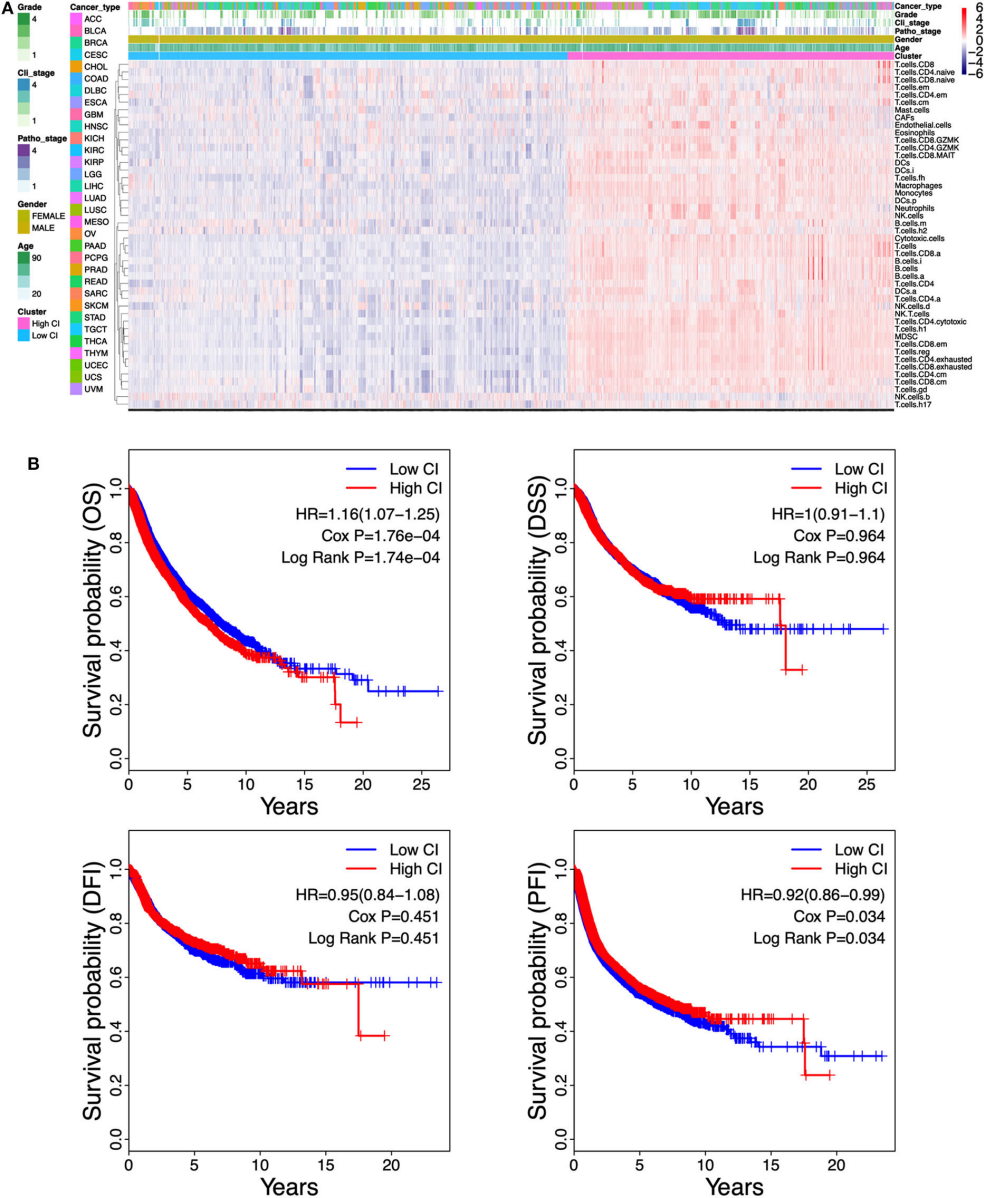
Correlation between the immune cell infiltration and survival in the pan-cancer cohort. **(A)** Unsupervised clustering separates The Cancer Genome Atlas (TCGA) pan-cancer cohort of 9,112 patients into two distinct immunophenotypes using the single-sample Gene Set Enrichment Analysis (ssGSEA) scores which represent the 46-cell infiltration. “Red color cluster” represents “hot” tumors with more immune cell infiltration, “blue color cluster” represents “cold” tumors with less immune cell infiltration. **(B)** Kaplan–Meier curves estimate the survival differences between the high cell infiltration cluster and the low cell infiltration cluster. Survival differences between the two clusters were detected by both Cox regression and log-rank methods. OS, overall survival; DSS, disease-specific survival; PFI, progression-free interval; DFI, disease-free interval.

Unsupervised clustering on the TCGA pan-cancer cohort (32 cancer types) showed that the tumor samples were predominantly separated into two clusters: low immune cell infiltration (Low CI) and high immune cell infiltration (High CI) (Figure 2A). In addition, patients in the High CI cluster had a worse overall survival (OS) (HR = 1.16, 95% CI = 1.07–1.25, Cox *P* = 1.76e−04, log-rank *P* = 1.74e−04) but a better progression-free interval (PFI) (HR = 0.92, 95% CI = 0.86-0.99, Cox *P* = 0.034, log-rank *P* = 0.034) compared to the Low CI cluster (Figure 2B).

Given the contradictory outcomes between OS and PFI in the pan-cancer cohort, we next performed unsupervised clustering on the individual cancer types. We found that each of these 32 cancer types could be divided into two clusters: Low CI or High CI cluster (Figure S4). Furthermore, immune cell infiltration was highly correlated with OS, PFI, or disease-specific survival (DSS) in 11 cancer types including adrenocortical carcinoma (ACC), cervical squamous cell carcinoma and endocervical adenocarcinoma (CESC), cholangiocarcinoma (CHOL), head and neck squamous cell carcinoma (HNSC), liver hepatocellular carcinoma (LIHC), LUAD, sarcoma (SARC), skin cutaneous melanoma (SKCM), glioblastoma multiforme (GBM), brain lower grade glioma (LGG), and uveal melanoma (UVM) (Figure S5). High CI indicated better prognosis in ACC, CESC, CHOL, HNSC, LIHC, LUAD, SARC, and SKCM (Figure S5A) but worse prognosis in GBM, LGG, and UVM (Figure S5B). In the other 22 cancer types, immune cell infiltration has no correlation with cancer prognosis (data not shown in Figure S5). Taken together, these results showed that immune cell infiltration results in heterogeneous prognostic outcomes in different cancer types.

### The Prognostic Relevance of Distinct Immune Cells Is Heterogeneous Across Different Cancer Types

**Figure 3:**
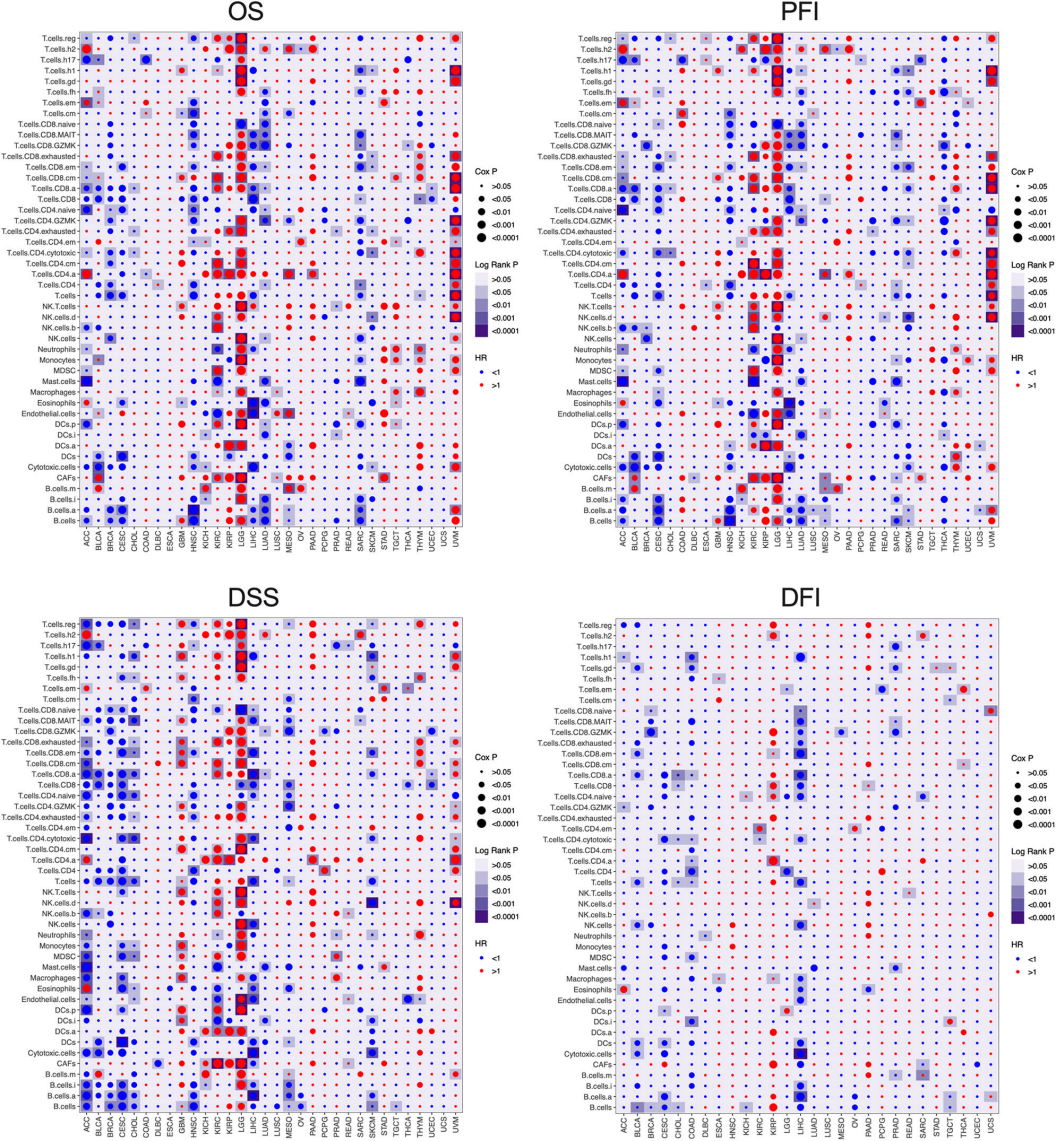
The prognosis value of the infiltrating immune cells in each cancer type. The association between the infiltrating immune cells and the survival of tumor patients was investigated using Cox regression and log-rank methods. The hazard ratio (HR) is <1.0, indicating a good effect on prognosis, and the HR value is greater than 1.0, indicating an adverse effect on prognosis. Cox P is represented by the size of points, and log-rank *P* is represented by the gradient of colors. *P* < 0.05 was used as the cutoff for significance. OS, overall survival; DSS, disease-specific survival; PFI, progression-free interval; DFI, disease-free interval.

To further understand the relationship between immune cell infiltration and tumor prognosis, the survival correlation of the 46 immune cells were analyzed in each cancer type. Overall, the correlation between infiltrating immune cells and the prognosis of cancer patients is consistent in OS, DSS, PFI, and disease-free interval (DFI). However, only a few immune cells are associated with DFI compared to OS, DSS, and PFI. Consistent with Figure S5, almost all the immune cell populations indicated a good prognosis in ACC, BRCA, CESC, HNSC, LIHC, LUAD, and SARC but a bad prognosis in GBM, LGG, and UVM (Figure 3). Among those cancer types where immune cell infiltration is associated with cancer prognosis, high B cell infiltration was correlated with good prognosis in most cancer types, except GBM, LGG, UVM, and kidney renal papillary cell carcinoma (KIRP). In addition to LGG, UVM, KIRC, and KIRP, high infiltration of T cells, CD8^+^ T cells, and active CD8^+^ T cells (T.cell.CD8.a) indicated a favorable prognosis in other cancer types. Infiltration of naive CD4^+^ T cells also led to a good outcome in various cancers, whereas that of activated CD4^+^ T cells (T.cell.CD4.a) indicated poor prognosis in most tumors. High infiltration of Th17 cells was associated with good prognosis in most cancer types, except for LGG and GBM, while that of Th2 cells portended worse prognosis in almost all tumors. In fact, the infiltration of all innate immunity-related cells, such as NK cells, myeloid-derived suppressor cells (MDSCs), macrophages, and DCs, was prognostically relevant in only a few tumors and varied considerably. The infiltrating regulatory T cells (Tregs) also showed a heterogeneous prognostic performance across more than a dozen tumors. Neutrophils, mast cells, and eosinophils were also related to the outcomes of several tumors, and high neutrophil infiltration predicted poor prognosis, whereas that of mast cells and eosinophils indicated a good prognosis. Finally, a high density of cancer-associated fibroblasts (CAFs) in the tumors was associated with adverse clinical outcomes in most tumors, while endothelial cells showed inconsistent prognostic performance across different tumors. Taken together, the infiltration of immune cell populations exhibited heterogeneous prognosis in different cancer types.

### The Immune-Cell Characteristic Score Model for Predicting Survival of Lung Adenocarcinoma

**Figure 4:**
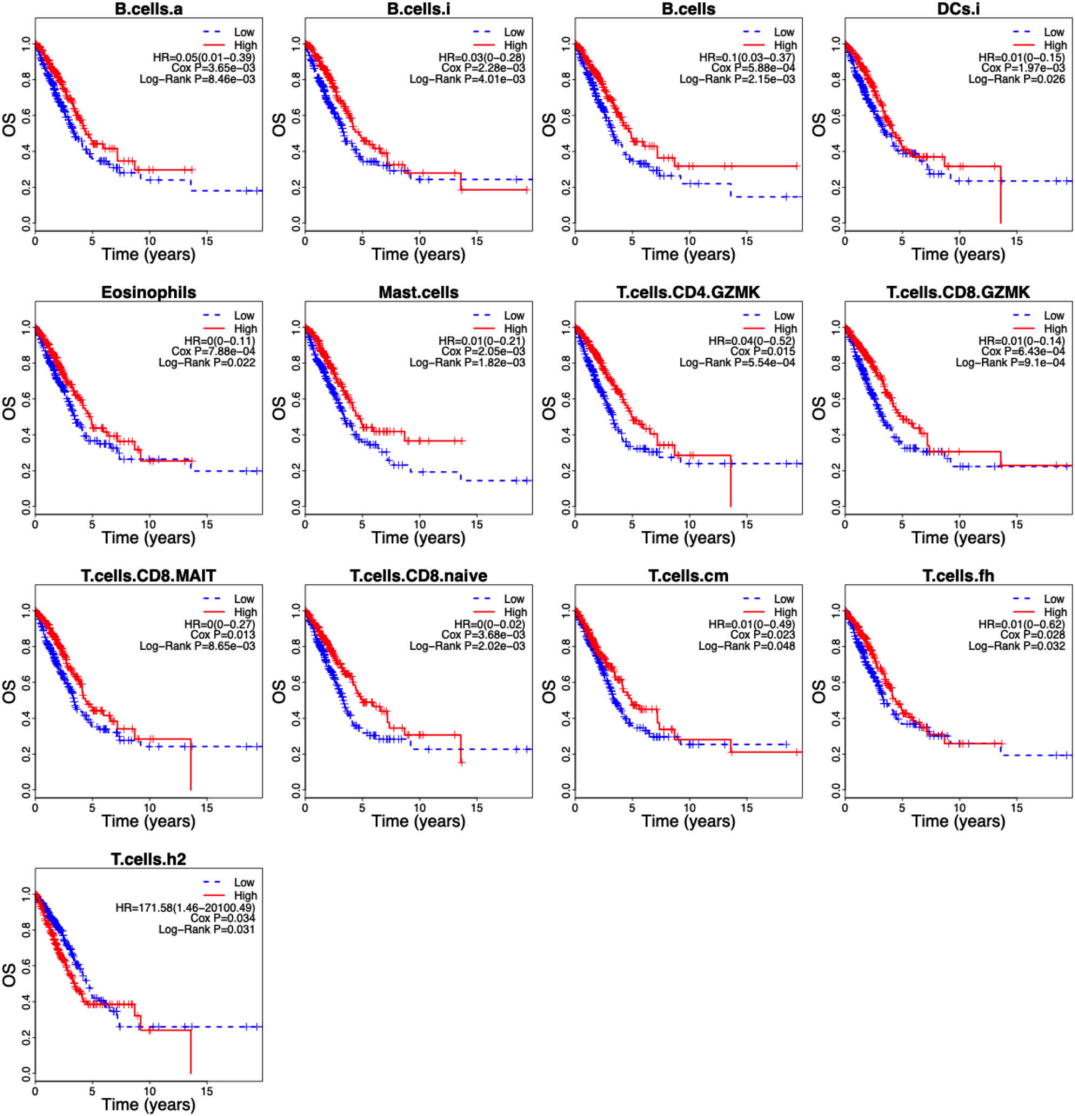
Kaplan–Meier curves of the 13 infiltrating immune cells in LUAD. The association between the infiltrating immune cells and the overall survival of LUAD patients was investigated using Cox regression and log-rank methods. Kaplan–Meier curves was drawn by the “survival” package based on R.

**Figure 5:**
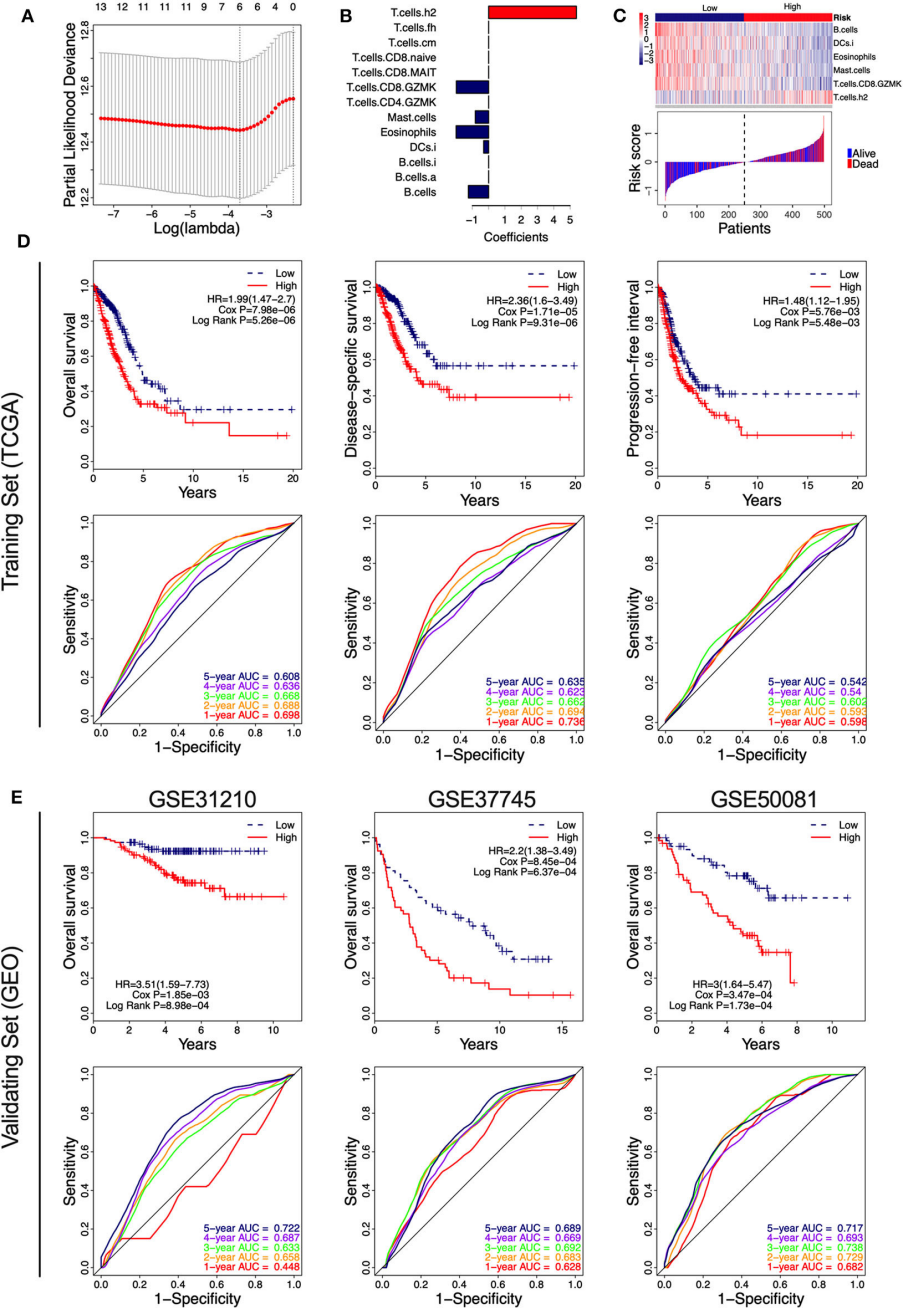
Identification of the immune-cell characteristic score (ICCS) and investigation of its prognostic value in lung adenocarcinoma (LUAD). **(A)** Cross-validation for tuning parameter selection in the least absolute shrinkage and selection operator (LASSO) model. **(B)** LASSO coefficient profiles of 13 prognosis-related immune cell populations. Variables whose LASSO coefficient is not equal to zero were used as candidate variables to construct the ICCS model. **(C)** The ICCS model classifies patients into low-ICCS and high-ICCS groups. **(D)** Kaplan–Meier curves and time-dependent receiver operating characteristic (ROC) curves of the prognostic ICCS model in the training set [The Cancer Genome Atlas (TCGA)]. The association between the ICCS and the survival of patients was investigated using Cox regression and log-rank methods. **(E)** Kaplan–Meier curves and time-dependent ROC curves of the prognostic ICCS model in the validating set [three Gene Expression Omnibus (GEO) datasets].

Of the 46 tumor infiltration immune cell populations, 13 cell populations including B cells (B.cells), activated B cells (B.cells.a), immature B cells (B.cells.i), immature DCs (DCs.i), eosinophils, mast cells (Mast.cells), granzyme K expressing CD4^+^ T cells (T.cells.CD4.GZMK), granzyme K expressing CD8^+^ T cells (T.cells.CD8.GZMK), mucosal-associated invariant CD8^+^ T cells (T.cells.CD8.MAIT), naive CD8^+^ T cells (T.cells.CD8.naive), central memory T cells (T.cells.cm), follicular helper T cells (T.cells.fh), and Type 2 T helper (Th2) cells (T.cells.h2) were correlated with the OS of LUAD patients (Figure 4). High infiltration of 12 immune cell populations indicated a favorable prognosis (HR <1, *P* < 0.05) and only high infiltration of the Th2 cells indicated an unfavorable prognosis (HR > 1, *P* < 0.05) of LUAD patients (Figure 4). By using LASSO regression analysis, six-cell populations including B cells, immature DCs, eosinophils, mast cells, granzyme K expressing CD8^+^ T cells, and Th2 cells were selected (Figures 5A,B). An ICCS model based on the selected cell populations was then constructed by calculating the ICCS of the patients, and the patients in each dataset were classified into low- and high-ICCS groups based on the median ICCS (Figure 5C). Heat map of the infiltration of the immune cells showed that high infiltration of the five cells (B cells, immature DCs, eosinophils, mast cells, granzyme K expressing CD8^+^ T cells) was involved in the low-ICCS group, whereas high infiltration of Th2 cells was involved in the high-ICCS group. Furthermore, there are more dead patients in the high-ICCS groups than in those in the low-ICCS groups (Figure 5C).

In the TCGA cohort, patients in the high-ICCS group had significantly shorter OS, DSS, and PFI compared to those in the low-ICCS group (Figure 5D). For the prediction of OS and DSS, the 1–5-year AUC values of the ROC curve were higher than 0.6, which showed a good survival prediction performance. For the prediction of PFI, all the 1–5-year AUC values of the ROC curve were lower than 0.6, which showed a limited survival prediction performance (Figure 5D). To investigate the reliability of the ICCS for prediction of OS of LUAD patients, the survival prediction performance of the ICCS was validated on three independent LUAD datasets from GEO (GSE31210, GSE37745, and GSE50081). As shown in Figure 5E, high-ICCS patients presenting significantly worse OS than low-ICCS patients (HR > 1, Cox *P* < 0.05, log-rank *P* < 0.05) in all the three datasets. Except for 1-year AUC values in GSE31210, the 1–5-year AUC values of the ROC curve were higher than 0.6 in all the three datasets. The 5-year AUC values were 0.722, 0.689, and 0.717 in the GSE31210, GSE37745, and GSE50081 datasets, respectively (Figure 5E). These data indicated that the ICCS effectively predicted the OS of LUAD patients and showed a reliable prediction performance across different LUAD datasets.

### The Immune-Cell Characteristic Score Is an Independent Prognostic Factor

To investigate the prognostic factor for LUAD patients, both univariate and multivariate Cox analyses were carried out based on the variables including the ICCS, age, gender, and pathologic stage. As shown in Table 1, age and gender were not associated with the OS of LUAD patients in all cohorts (*P* > 0.05). In the TCGA cohort, both high ICCS and the pathologic stages II–IV were identified as independent unfavorable prognostic factors (HR > 1, *P* < 0.05). The association between the ICCS and the OS was also confirmed in three GEO cohorts (HR > 1, *P* < 0.05). Stage II was confirmed as an independent unfavorable prognostic factor in GSE31210 and GSE50081 (HR > 1, *P* < 0.05), and stage III was confirmed as an independent unfavorable prognostic factor in GSE37745 (HR > 1, *P* < 0.05).

**Table 1:** Cox regression analysis of the ICCS, clinicopathological features, and overall survival of LUAD patients.

**Variables**	**Group**	**Patients (*N*)**	**Univariate analysis**		**Patients (*N*)**	**Multivariate analysis**	
			**HR (95% CI)**	***P***		**HR (95% CI)**	***P***
**TCGA**							
ICCS	Low/High	249/249	1.99 (1.47–2.70)	7.98E-06	244/244	1.97 (1.44–2.69)	2.31E-05
Age	≤60/>60	153/335	1.13 (0.82–1.56)	4.60E-01	153/335	1.21 (0.88–1.68)	2.43E-01
Gender	Female/Male	268/230	1.04 (0.78–1.40)	7.70E-01	264/224	0.97 (0.72–1.31)	8.39E-01
Pathologic stage	I/II	271/120	2.42 (1.68–3.48)	1.95E-06	264/118	2.16 (1.49–3.13)	5.23E-05
Pathologic stage	I/III	271/81	3.58 (2.45–5.24)	4.83E-11	264/80	3.37 (2.29–4.94)	5.91E-10
Pathologic stage	I/IV	271/26	3.83 (2.21–6.64)	1.82E-06	264/26	3.04 (1.73–5.35)	1.10E-04
**GSE31210**							
ICCS	Low/High	113/113	3.51 (1.59–7.73)	1.85E-03	113/113	3.09 (1.4–6.85)	5.38E-03
Age	≤60/>60	108/118	1.27 (0.65–2.48)	4.86E-01	108/118	1.44 (0.74–2.82)	2.87E-01
Gender	Female/Male	121/105	1.52 (0.78–2.96)	2.19E-01	121/105	1.25 (0.64–2.45)	5.13E-01
Pathologic stage	I/II	168/58	4.23 (2.17–8.24)	2.17E-05	168/58	3.78 (1.93–7.41)	1.09E-04
**GSE37745**							
ICCS	Low/High	53/53	2.20 (1.38–3.49)	8.45E-04	53/53	2.32 (1.4–3.85)	1.14E-03
Age	≤60/>60	46/60	1.22 (0.77–1.93)	3.98E-01	46/60	1.39 (0.86–2.24)	1.81E-01
Gender	Female/Male	60/46	1.26 (0.80–1.97)	3.16E-01	60/46	1.04 (0.64–1.69)	8.66E-01
Pathologic stage	I/II	70/19	1.47 (0.83–2.60)	1.84E-01	70/19	1.46 (0.83–2.60)	1.92E-01
Pathologic stage	I/III	70/13	2.10 (1.11–3.99)	2.34E-02	70/13	1.94 (1.01–3.74)	4.62E-02
Pathologic stage	I/IV	70/4	1.53 (0.47–4.93)	4.79E-01	70/4	0.86 (0.25–2.90)	8.06E-01
**GSE50081**							
ICCS	Low/High	64/64	3.00 (1.64–5.47)	3.47E-04	64/64	3.37 (1.83–6.18)	9.10E-05
Age	≤60/>60	19/109	1.50 (0.64–3.51)	3.54E-01	19/109	1.76 (0.74–4.16)	2.00E-01
Gender	Female/Male	63/65	1.35 (0.78–2.34)	2.85E-01	63/65	1.57 (0.89–2.75)	1.16E-01
Pathologic stage	I/II	92/36	2.53 (1.45–4.44)	1.16E-03	92/36	2.72 (1.55–4.77)	5.14E-04

*HR, hazard ratio; CI, confidence interval; ICCS, immune-cell characteristic score; LUAD, lung adenocarcinoma; TCGA, The Cancer Genome Atlas*.

**Figure 6:**
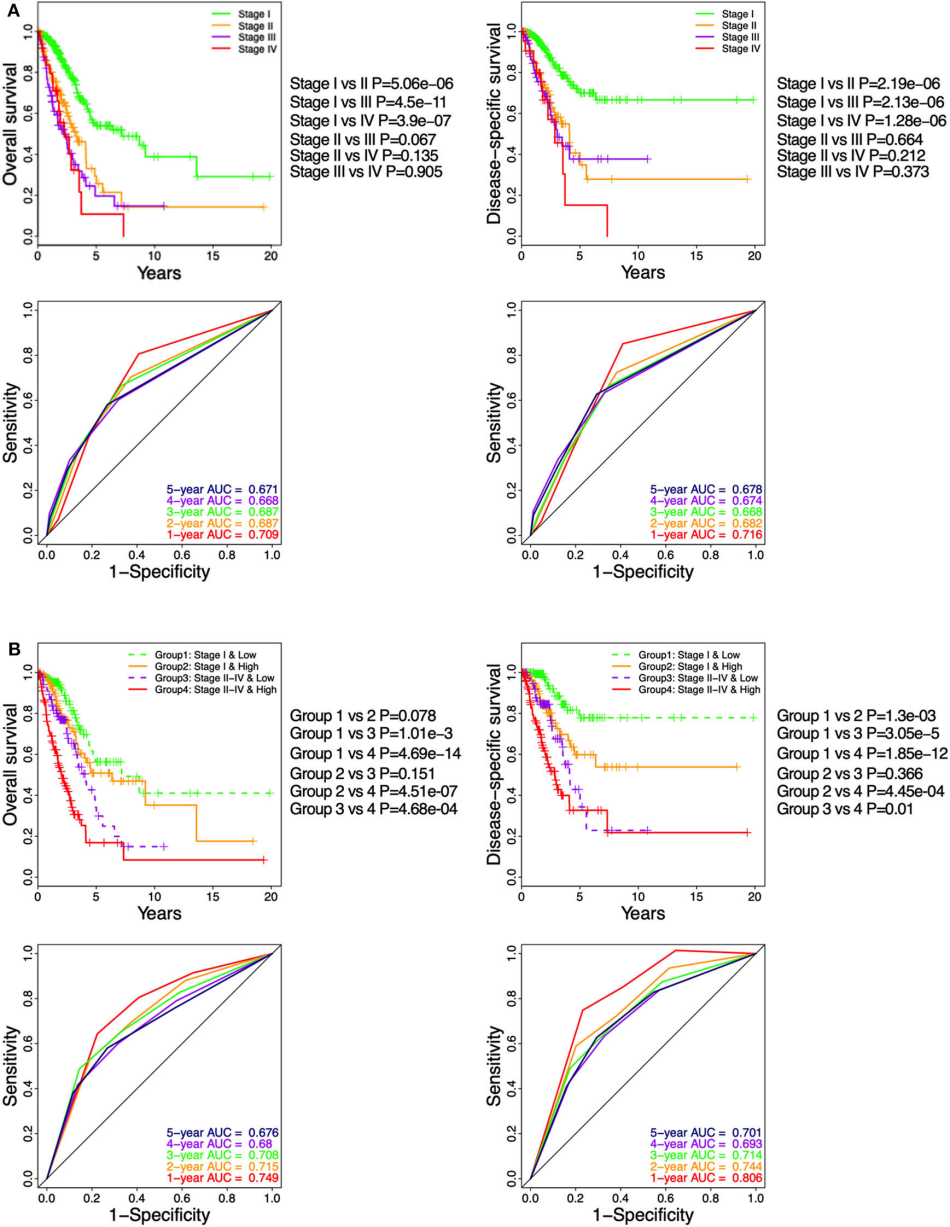
Stratification analysis on The Cancer Genome Atlas (TCGA) cohort based on immune-cell characteristic score (ICCS) and pathologic stage. **(A)** Kaplan–Meier analysis and time-dependent receiver operating characteristic (ROC) curves show the prognostic values of the pathologic stage using the TCGA cohort. **(B)** Kaplan–Meier analysis and time-dependent ROC curves present the prognostic values for patients grouped by combining the stage and the ICCS.

In TGCA cohort, Kaplan–Meier curve showed that LUAD patients at pathologic stages II–IV (Figure 6A) had similar OS and DSS (log-rank *P* > 0.05), which were significantly shorter compared to that of the stage I patients (log-rank *P* < 0.05; Figure 6A). The time-dependent ROC curve showed that the pathological stage achieved 5-year AUC values of 0.671 and 0.678 for OS and DSS, respectively (Figure 6A), indicating a competent predictive performance. Finally, the patients were stratified based on their pathological stage (I and II–IV) and then further classified into the low-ICCS and high-ICCS groups. The ICCS predicted the DSS (log-rank *P* = 1.3e−03) but not the OS (log-rank *P* = 0.078) for patients in stage I (Figure 6B). In addition, there were no significant differences in the OS (log-rank *P* = 0.151) and DSS (log-rank *P* = 0.366) between the high-ICCS patients in stage I and the low-ICCS patients in stage II–IV. However, among the patients in stages II–IV, the high-ICCS group showed significantly shorter OS (log-rank *P* = 4.68e−04) and DSS (log-rank *P* = 0.01) compared to the low-ICCS group (Figure 6B). Taken together, the ICCS is independent of the pathological stage for predicting OS and DSS in LUAD patients.

## Discussion

The TME is a complex ecosystem composed of malignant, stromal, and immune cells. The tumor-infiltrating immune cells are a critical player in tumor progression and immunotherapeutic response (29). The composition of the tumor-infiltrating population reflects the mechanisms underlying anticancer immune responses and can help identify novel prognostic signatures. Commonly used methods for identifying tumor immune cell infiltration mainly rely on immunohistochemistry (IHC) and flow cytometry. These methods are limited by many factors, including the amount of tumor tissue required and the number of cell types that can be measured simultaneously (30). The computational method applied to the gene expression profile of bulk tumors provides another option for evaluating the immune status within tumor tissues (3, 31). The computational method can also integrate multiple gene expression datasets for analysis, providing the analysis results of a larger number of samples. There are currently two commonly used computational methods, one is ssGSEA, and the other is the deconvolution method, such as CIBERSORT (31). The ssGSEA method ranks marker genes by integrating the differences between the empirical cumulative distribution of those genes based on their absolute expression in a single sample and is widely used for sample level enrichment analysis (16). CIBERSORT was originally developed and verified using microarray data (32). This method requires that the input data are Gaussian distribution, while the unnormalized RNA-seq count is negative binomial distribution (32). Therefore, when analyzing RNA sequencing data, it must be converted into “microarray-like” data before it can be used for subsequent analysis (33). However, ssGSEA does not require data conversion when analyzing RNA sequencing data. Furthermore, CIBERSORT can only estimate the proportion of 22 cell types, while ssGSEA can estimate more cell types which are determined based on the number of gene sets.

In this study, we collected gene sets that can represent immune cells from six published articles (3, 22–26). Through correlation analysis between immune cells and consistency analysis with traditional markers, 46-cell gene sets were finally screened. Subsequently, we used the ssGSEA to characterize and quantify the tumor-infiltrating immune cells from their gene expression data across multiple cancers. Among the 32 types of cancer, 9,112 individual tumor samples can be divided into two clusters: High CI and Low CI phenotypes, which can be interpreted as “hot” and “cold” tumors (34). High CI was associated with better prognosis in ACC, CESC, CHOL, HNSC, LIHC, LUAD, SARC, and SKCM and worse prognosis in GBM, LGG, and UVM, indicating a heterogeneous prognostic outcome depending on the cancer type. Interestingly, for patients with different grades of gliomas (LGG and GBM), High CI is always associated with a poor prognosis, indicating that the treatment of gliomas by promoting the infiltration of immune cells may have the opposite effect. In contrast, for “cold” tumors with little or no immune cell infiltration that are usually correlated with a bad prognosis, modifying a “cold” tumor into a “hot” tumor may sensitize the patient to immunotherapy.

The TME harbors both immune-suppressive and activating cells, and the tumor infiltrates are highly heterogeneous depending on the specific cancer type or the tumor model. T-cell infiltration is a reliable predictor of patient outcome and has been implemented in treating various cancers (35). Studies have confirmed the positive impact of T cells in tumor progression (7), and their exclusion from the TME leads to immune privilege (36). In the present study, we found that exclusion of CD8^+^ and CD4^+^ T cells were only associated with the prognosis of a few tumors and showed inconsistent performance. Nevertheless, the high infiltration of CD8^+^ T cells and active CD8^+^ T cells indicated a good prognosis, suggesting a therapeutic advantage of activating these cells in the TME. Immunosuppressive cells, such as tumor-associated macrophages (TAMs) and MDSCs, have a significant bearing on the survival of LUAD patients (37, 38). We found that the infiltration of macrophages was correlated to the survival of a few tumors, with an inconsistent predictive performance for OS, DSS, and PFI. Th2 cells have an immunoregulatory role in tumor growth and can induce tumor cell necrosis by secreting type 2 cytokines within the TME (39, 40). Furthermore, the Th2 inflammatory cytokine IL-33 is associated with poor prognosis in lung cancer patients (41). In this study, we found that although Th2 is only associated with the prognosis of patients with ACC, kidney chromophobe (KICH), KIRP, LUAD, and pancreatic adenocarcinoma (PAAD), it showed an adverse prognostic factor in all these cancer types. B cells are key players in the immune system that modulate T-cell responses by providing antigens and secreting cytokines (42). In the present study, high B-cell infiltration indicated a good prognosis in most cancer types, except GBM, LGG, UVM, and KIRP. Our results are consistent with previous evidence supporting the role of tumor-infiltrating lymphocytes in mediating immunotherapeutic responses.

Non-small-cell lung cancer (NSCLC) and melanoma are two cancer types that respond to immunotherapy largely due to the high mutation burden of these tumors (43), which have been proposed to associate with tumor immune infiltration (44). In the present study, we investigated the ssGSEA score which represents the infiltrating immune cells in different cancer types and found that most immune cells can obtain a relatively high ssGSEA score in LUAD, indicating that LUAD may be a “hot” tumor type. We also found that High CI was associated with better OS and DSS in LUAD patients but had no significant effect on PFI compared to Low CI. We speculate that this is mainly because not all the cells show a consistent prognostic correlation in the same cluster. Thus, classifying patients into two immunophenotypic clusters may not be the optimal prognostic tool. For instance, high Th2 cell infiltration was correlated with poor prognosis in LUAD patients. This study is consistent with previously reported results that high levels of Th2 cells are associated with poor prognosis of clear cell renal cell carcinoma (30). In order to better display the survival difference of patients with different immune cell infiltration status, we established an ICCS model based on six survival-related immune cells. In the ICCS model, high infiltration of B cells, immature DCs, eosinophils, mast cells, granzyme K expressing CD8^+^ T cells were involved in the low-ICCS group, corresponding to a favorable prognosis, whereas high infiltration of Th2 cells was involved in the high-ICCS group, corresponding to an unfavorable prognosis. When regrouping with the ICCS, all of the OS, DSS, and PFI showed differences between the low- and high-ICCS groups, suggesting that ICCS is a better classification method than unsupervised clustering. Because high infiltration of Th2 cells is related to unfavorable prognosis, we suspect that blocking the activation of Th2 cells might prolong the survival of high-ICCS LUAD patients.

The pathological stage of the tumor is a traditional prognostic factor for LUAD patients (45). In this study, we confirmed that the advanced pathologic stages II, III, and IV were adverse prognostic factors. However, patients with stages II, III, and IV have no significant difference in survival, indicating that the pathological stage has certain defects in predicting the survival of LUAD patients. Due to the heterogeneity of TME, the survival rate of tumor patients at the same pathologic stage may be significantly different (46). By using the ICCS model, patients divided into the high-ICCS group showed worse survival compared to the low-ICCS group regardless of the tumor stage. This could overcome the prognostic limitations of pathologic stage and further stratified the stage I and stage II–IV patients into the low- and high-ICCS groups. Furthermore, since the ICCS is based on the infiltration of immune cells, the high-ICCS patients may also benefit from treatments targeting certain immune cells.

## Conclusions

This study evaluated the landscape of intra-tumoral immune cells in 32 cancer types and observed considerable heterogeneity in the prognostic relevance of these tumor-infiltrating immune cells in different cancer types. In LUAD, the infiltration of 12 immune cells was associated with a favorable prognosis and that of Th2 cells with unfavorable outcomes. An ICCS model was constructed, and the ICCS is an independent prognostic factor of LUAD. The intra-tumoral immune cells can deepen our understanding of the TME of LUAD and can have implications for immunotherapy.

## Data Availability Statement

Publicly available datasets were analyzed in this study. This data can be found here: TCGA, GSE31210, GSE37745, GSE50081.

## Author Contributions

SZ, SW, and MW contributed to downloading the data, performed the analysis, and wrote the manuscript. JD and JW conceived and designed the study and contributed to the critical review of the manuscript. All authors read and approved the final manuscript.

## Conflict of Interest

The authors declare that the research was conducted in the absence of any commercial or financial relationships that could be construed as a potential conflict of interest.
