# Supplementary material for: Pan-Cancer Analysis of Immune Cell Infiltration Identifies a Prognostic Immune-Cell Characteristic Score (ICCS) in Lung Adenocarcinoma
Source: Front Immunol. 2020 Jun 30;11:1218. doi: 10.3389/fimmu.2020.01218 (PMC7344231; doi:10.3389/fimmu.2020.01218)
Supplement: Supplementary file 1 [file Data_Sheet_1.pdf]

Supplementary Material

Table S1 The sample number of RNA-seq gene expression data from TCGA dataset

Cohort	Abbreviation	Sample number
Adrenocortical carcinoma	ACC	79
Bladder Urothelial Carcinoma	BLCA	426
Breast invasive carcinoma	BRCA	1218
Cervical squamous cell carcinoma and endocervical adenocarcinoma	CESC	308
Cholangiocarcinoma	CHOL	45
Colon adenocarcinoma	COAD	193
Lymphoid Neoplasm Diffuse Large B-cell Lymphoma	DLBC	48
Esophageal carcinoma	ESCA	196
Glioblastoma multiforme	GBM	172
Head and Neck squamous cell carcinoma	HNSC	566
Kidney Chromophobe	KICH	91
Kidney renal clear cell carcinoma	KIRC	606
Kidney renal papillary cell carcinoma	KIRP	323
Brain Lower Grade Glioma	LGG	530
Liver hepatocellular carcinoma	LIHC	423
Lung adenocarcinoma	LUAD	576
Lung squamous cell carcinoma	LUSC	553
Mesothelioma	MESO	87
Ovarian serous cystadenocarcinoma	OV	308
Pancreatic adenocarcinoma	PAAD	183
Pheochromocytoma and Paraganglioma	PCPG	187
Prostate adenocarcinoma	PRAD	550
Rectum adenocarcinoma	READ	105
Sarcoma	SARC	265
Skin Cutaneous Melanoma	SKCM	474
Stomach adenocarcinoma	STAD	450
Testicular Germ Cell Tumors	TGCT	156
Thyroid carcinoma	THCA	572
Thymoma	THYM	122
Uterine Corpus Endometrial Carcinoma	UCEC	201
Uterine Carcinosarcoma	UCS	57
Uveal Melanoma	UVM	80

Table S2. The known markers of cell types

Cell type Abbreviation	Cell type	Markers
B.cells	B cells	CD19, MS4A1, CD79A, CD79B
CAFs	Cancer associated fibroblasts	FAP, THY1, COL1A1, COL3A1
DCs.a	Activated Dendritic cells	CCL1, CD83, LAMP3
DCs.i	Immature dendritic cells	CD1A, CD1E
Endothelial.cells	Endothelial cells	VWF, CDH5, SELE
Eosinophils	Eosinophils	IL3RA, IL5RA, CSF2RA
Macrophages	Macrophages	CD14, CD68, CD163, CSF1R, FCGR3A
Mast.cells	Mast cells	FCER1G, CMA1, MS4A2, TPSAB1
Monocytes	Monocytes	CD14, FCGR3A
Neutrophils	Neutrophils	ITGAM, FUT4, FCGR3A, ITGA4, CEACAM8
NK.cells	Natural killer cells	PRF1, GZMA, GZMB
T.cells	T lymphocytes	CD3E

Table S3. Gene sets of immune cells for single sample Gene Set Enrichment Analysis (ssGSEA)

Cell type	Cell type Abbreviation	Markers
B cells	B.cells	B.cells, NA, BANK1, CD79A, CD79B, FCER2, FCRL2, FCRL5, MS4A1, PAX5, POU2AF1, STAP1, TCL1A
Activated B cells	B.cells.a	B.cells.a, NA, ADAM28, CD180, CD79B, BLK, CD19, MS4A1, TNFRSF17, GNG7, MICAL3, SPIB, HLA-DOB, PNOC, FCRL2, BACH2, CR2, TCL1A, AKNA, ARHGAP25, CCL21, CD27, CD38, CLEC17A, CLEC9A, CLECL1
Immature B cells	B.cells.i	B.cells.i, NA, CD22, CYBB, FAM129C, FCRL1, FCRL3, FCRL5, FCRLA, HDAC9, HLA-DQA1, HVCN1, KIAA0226, NCF1, NCF1B, P2RY10, SP100, TXNIP, STAP1, TAGAP, ZCCHC2
Matured B cells	B.cells.m	B.cells.m, NA, AICDA, CCNA2, CDKN3, CLCN5, ENPP1, FCER1A, FCRL4, MYC, RUNX2, SORL1, SOX5, STAT5A, STAT5B, TLR9
Cancer associated fibroblasts	CAFs	CAFs, NA, FAP, THY1, DCN, COL1A1, COL1A2, COL6A1, COL6A2, COL6A3, CXCL14, LUM, COL3A1, DPT, ISLR, PODN, CD248, FGF7, MXRA8, PDGFRL, COL14A1, MFAP5, MEG3, SULF1, AOX1, SVEP1, LPAR1, PDGFRB, TAGLN, IGFBP6, FBLN1, CA12, SPOCK1, TPM2, THBS2, FBLN5, TMEM119, ADAM33, PRRX1, PCOLCE, IGF2, GFPT2, PDGFRA, CRISPLD2, CPE, F3, MFAP4, C1S, PTGIS, LOX, CYP1B1, CLDN11, SERPINF1, OLFML3, COL5A2, ACTA2, MSC, VASN, ABI3BP, C1R, ANTXR1, MGST1, C3, PALLD, FBN1, CPXM1, CYBRD1, IGFBP5, PRELP, PAPSS2, MMP2, CKAP4, CCDC80, ADAMTS2, TPM1, PCSK5, ELN, CXCL12, OLFML2B, PLAC9, RCN3, LTBP2, NID2, SCARA3, AMOTL2, TPST1, LOC399959, CTGF, RARRES2, FHL2
Cytotoxic cells	Cytotoxic.cells	Cytotoxic.cells, NA, GNLY, CTSW, DUSP2, KLRK1, GZMH, GZMA, APBA2, WHAMML1, KLRB1, KLRD1, NKG7, KLRF1, SIGIRR, RORA, ZBTB16, APOL3, RUNX3
Dendritic cells	DCs	DCs, NA, CD209, HSD11B1, NPR1, CCL13, CCL17, CCL22, PPFIBP2
Activated Dendritic cells	DCs.a	DCs.a, NA, EBI3, LAMP3, IDO1, OAS3, CCL1
Immature dendritic cells	DCs.i	DCs.i, NA, CLEC10A, CSF1R, CTNS, F13A1, FABP4, VASH1, SLC7A8, FZD2, GSTT1, GUCA1A, TACSTD2, MMP12, SYT17, NUDT9, PPARG, PREP, RAP1GAP, CARD9, MS4A6A, BLVRB, SLC26A6, TM7SF4, LMAN2L, PDXK, CH25H, CD1A, CD1B, CD1C, CD1E, ABCG2, HS3ST2
Plasmacytoid dendritic cells	DCs.p	DCs.p, NA, CBX6, DAB2, DDX17, HIGD1A, IDH3A, IL3RA, MAGED1, NUCB2, OFD1, OGT, PDIA4, SERTAD2, SIRPA, TMED2, ENG, FCAR, IGF1, ITGA2B, GABARAP, GPX1, KRT23, PROK2, RALB, RETNLB, RNF141, SEC14L1, SEPX1, EMP3, CD300LF, ABTB1, KLHL21, PHRF1
Endothelial cells	Endothelial.cells	Endothelial.cells, NA, PECAM1, VWF, CDH5, CLDN5, PLVAP, ECSCR, SLCO2A1, CCL14, MMRN1, MYCT1, KDR, TM4SF18, TIE1, ERG, FABP4, SDPR, HYAL2, FLT4, EGFL7, ESAM, CXorf36, TEK, TSPAN18, EMCN, MMRN2, ELTD1, PDE2A, NOS3, ROBO4, APOLD1, PTPRB, RHOJ, RAMP2, GPR116, F2RL3, JUP, CCBP2, GPR146, RGS16, TSPAN7, RAMP3, PLA2G4C, TGM2, LDB2, PRCP, ID1, SMAD1, AFAP1L1, ELK3, ANGPT2, LYVE1, ARHGAP29, IL3RA, ADCY4, TFPI, TNFAIP1, SYT15, DYSF, PODXL, SEMA3A, DOCK9, F8, NPDC1, TSPAN15, CD34, THBD, ITGB4, RASA4, COL4A1, ECE1, GFOD2, EFNA1, PVRL2, GNG11, HERC2P2, MALL,

		PPM1F, PKP4, LIMS3, CD9, RAI14, ZNF521, RGL2, HSPG2, TGFBR2, RBP1, FXYD6, MATN2, S1PR1, FAM38A, PDGFA, ADAM15, HAPLN3, APP
Eosinophils	Eosinophils	Eosinophils, NA, GIPR, LRMP, FOSB, RRP12, GPR183, NR4A3, ST3GAL6, DEPDC5, PDE6C, PKD2L2, GPR65, IL5RA, P2RY14, DACH1, DAPK2, EMR3
Macrophages	Macrophages	Macrophages, NA, APOC1, C1QC, CD14, CD163, CD300C, CD300E, CSF1R, F13A1, FPR3, HAMP, IL1B, LILRB4, MS4A6A, MSR1, SIGLEC1, VSIG4
Mast cells	Mast.cells	Mast.cells, NA, ABCC4, CEACAM8, ADCYAP1, CMA1, CPA3, CTSG, NR0B1, ELANE, MS4A2, GATA2, HPGDS, HDC, HPGD, LOC339524, KIT, VWA5A, MAOB, MPO, PRG2, SLC24A3, PTGS1, PPM1H, TPSB2, SLC18A2, TAL1, TPSAB1, SCG2, MLPH, CALB2, SIGLEC6
Myeloid-derived suppressor cells	MDSC	MDSC, NA, CCR2, CD14, CD2, CD86, CXCR4, FCGR2A, FCGR2B, FCGR3A, FERMT3, GPSM3, IL18BP, IL4R, ITGAL, ITGAM, PARVG, PSAP, PTGER2, PTGES2, S100A8, S100A9
Monocytes	Monocytes	Monocytes, NA, CD33, CD300C, CD300E, CECR1, CLEC6A, CPVL, EGR2, EREG, MS4A6A, NAGA, SLC37A2
Neutrophils	Neutrophils	Neutrophils, NA, LILRB2, CEACAM3, HPSE, CSF3R, FCAR, FCGR3B, CD93, FPR1, FPR2, ALPL, CXCR1, CXCR2, KCNJ15, CYP4F3, MME, G0S2, SLC25A37, PDE4B, CPPED1, VNN3, S100A12, SLC22A4, BST1, DYSF, HIST1H2BC, CRISPLD2, SIGLEC5, TNFRSF10C, MGAM, CREB5, TECPR2
Natural killer cells	NK.cells	NK.cells, NA, AKT3, AXL, BST2, CDH2, CRTAM, CSF2RA, CTSZ, CXCL1, CYTH1, DAXX, DGKH, DLL4, DPYD, ERBB3, F11R, FAM27A, FAM49A, FASLG, FCGR1A, FN1, FSTL1, FUCA1, GBP3, GLS2, GRB2, LST1, BCL2, CDC5L, FGF18, FUT5, FZR1, GAGE2C, IGFBP5, KANK2, LDB3
CD56bright natural killer cells	NK.cells.b	NK.cells.b, NA, MPPED1, DUSP4, FOXJ1, RRAD, XCL1, PLA2G6, MADD, MARCH6, TRAPPC9, LPCAT4
CD56dim natural killer cells	NK.cells.d	NK.cells.d, NA, GTF3C1, GZMB, KIR2DL3, KIR2DS1, KIR2DS5, KIR3DL1, KIR3DL2, KIR3DS1, SPON2, IL21R, S1PR5, TTC38, PMEPA1, KIR3DL3
Natural killer T cells	NK.T.cells	NK.T.cells, NA, BTN2A2, CD101, CD109, CNPY3, CNPY4, CREB1, CRTC2, CRTC3, CSF2, KLRC1, FUT4, ICAM2, IL32, LAMP2, LILRB5, KLRG1, HSPA4, HSPB6, ISM2, ITIH2, KDM4C, KIR2DS4, KIRREL3, SDCBP, NFATC2IP, MICB, KIR2DL1, KIR2DL3, KIR3DL1, KIR3DL2, NCR1, FOSL1, TSLP, SLC7A7, SPP1, TREM2, UBASH3A, YBX2, CCDC88A, CLEC1A, THBD, PDPN, VCAM1, EMR1
T lymphocytes	T.cells	T.cells, NA, CD2, CD3D, CD3E, CD3G, CD8A, SIRPG, TIGIT, GZMK, ITK, SH2D1A, CD247, PRF1, NKG7, IL2RB, SH2D2A, KLRK1, ZAP70, CD7, CST7, LAT, PYHIN1, SLA2, STAT4, CD6, CCL5, CD96, TC2N, FYN, LCK, TCF7, TOX, IL32, SPOCK2, SKAP1, CD28, CBLB, APOBEC3G, PRDM1
CD4+ T cells	T.cells.CD4	T.cells.CD4, NA, ANKRD55, DGKA, FOXP3, GCNT4, IL2RA, MDS2, RCAN3, TBC1D4, TRAT1
Activated CD4+ T cells	T.cells.CD4.a	T.cells.CD4.a, NA, AIM2, BIRC3, BRIP1, CCL20, CCL4, CCL5, CCNB1, CCR7, DUSP2, ESCO2, ETS1, EXO1, EXOC6, IARS, ITK, KIF11, KNTC1, NUF2, PRC1, PSAT1, RGS1, RTKN2, SAMSN1, SELL, TRAT1
Central memory CD4+ T cells	T.cells.CD4.cm	T.cells.CD4.cm, NA, ABHD3, AHNAK, ANXA2P2, AQP3, ATHL1, BMI1, BZW2, CD63, COL4A1, CYLD, ELMO2, FYN, GLIPR1, GSS, IFITM2, ITGB1, ITGB2, KLF5, LSP1, NDUFB9, PKM2, SFXN3, SIRPG, SMAD4, STX4, TRADD, VIM, XRCC6

cytotoxic memory CD4+ T cells	T.cells.CD4.cytotoxic	T.cells.CD4.cytotoxic , NA, FGFBP2, CX3CR1, GNLY, GZMB, GZMA, CCL5, NKG7, GZMH, S1PR5, FGR, ADGRG1, CST7, C1orf21, PRSS23, PLEK, ZEB2, TARP, KLRG1, FCRL6, SLAMF7, ADRB2, ZEB2-AS1, PRF1, CCL4, HOPX, A2M, CTSW, SPON2, EFHD2, CD300A, MYBL1, TGFBR3, SAMD3, ZNF683, LINC00612, PZP, FCGR3A, TM4SF19, ADGRG5, PXN, LILRB1, MYO1F, C12orf75, AHNAK, PTGDS, LGR6, RUNX3, OSBPL5, VCL, SYNE1, ABI3, MBP, PLEKHG3, LYAR, PTPN4, TTC16, CHST12, GZMM, LITAF, SH3BP5, PROK2, PTCH1, CEP78, PATL2, CD244, AKR1C3, RASSF1, LPCAT1, GLUL, PLEKHA1, TM4SF19-TCTEX1D2, AGPAT4, TTC38, LLGL2, SUN2, RAP2B, APMAP, LOC100130872, GSTP1, GK5, SYNE2, DSTN, RAP1B, SPN, SLC2A1, RAB29, TMEM181, ABCA2, ARL4C, ARL5A, FUT11, PAFAH2, NPRL2
Effector memory CD4+ T cells	T.cells.CD4.em	T.cells.CD4.em, NA, ATM, CASP3, CASQ1, CD300E, DARS, DOCK9, EXOSC9, EZH2, GDE1, IL34, NCOA4, NEFL, PDGFRL, PTGS1, REPS1, SCG2, SDPR, SIGLEC14, SIGLEC6, TAL1, TFEC, TIPIN, TPK1, UQCRB, USP9Y, WIPF1, ZCRB1
Exhausted CD4+ T cells	T.cells.CD4.exhausted	T.cells.CD4.exhausted, NA, TNFRSF18, TIGIT, CTLA4, RGS1, DUSP4, ENTPD1, CREM, BATF, SRGN, ICOS, CXCL13, GAPDH, DNPH1, PTTG1, F2R, HAVCR2, ASB2, PDCD1, PHTF2, SIRPG, NDFIP2, MAF, ETV7, LYST, IL21, ADAM19, TOX, TOX2, CDKN2A, ZBED2, PDE7B, CD82, TNFSF8, UNQ6494, PKM, CTSD, RBPJ, EPSTI1, AKAP5, RAB11FIP1, BHLHE40-AS1, FKBP1A-SDCBP2, IFNG, TRPS1, ITM2A, CCDC50, RHBDD2, BST2, IGFLR1, RNF19A, LINC00963, GNG4, SPOCK2, MAP1LC3A, GALM, NAB1, IKZF3, IFI27L2, NR3C1, PRKAR1A, LASP1, TRAF3, AHI1, TBXAS1, PHEX, SARDH, LAP3, TPI1, GBP4, CKAP2, CAMK1, ATXN1, ITGA2, RGS2, METTL8, MIR155HG, SLC6A6, BTLA, SQRDL, TMEM2, CD38, MAPKAPK3, PSME2, KCNK5, UBE2L6, LIMA1, SHFM1, C9orf16, SUSD6, CHN1, ST8SIA1, WARS, LIMS1, RABGAP1L, ARHGAP9, ATP6V1A, TBC1D2B, CLTC, IFI35, FUT8, OGG1, SLC9A9, WSB2, RILPL2, SMC5, HNRNPK, DBNL, DR1, CAPZB, CD84, NUTF2, ATP5C1, AKR1A1, ZEB1, PRKAG1, GSPT1, SYNJ1, PTPN11, N4BP2, SFXN1, NMI, UQCRH
Granzyme K expressing CD4+ T cells	T.cells.CD4.GZMK	T.cells.CD4.GZMK, NA, GZMK, CD69, DPP4, PARP8, DUSP1, IFNGR1, SLC4A10, ABCB1, PVRIG, CAPG
Naïve CD4+ T cells	T.cells.CD4.naive	T.cells.CD4.naive, NA, CCR7, TCF7, TXK, GIMAP5, MAL, MYC, IL7R, ABLIM1, LDLRAP1, FHIT, TRABD2A, C1orf162, NOSIP, EEF1B2, SERINC5, GPR183, ACTN1, SATB1, TSHZ2, CD55, PRKCA, RPS5, TMEM204, SLC40A1, NELL2, SVIL, GIMAP8, LRRN3, TESPA1, LRRC75A-AS1, FAM117B, EIF3L, C6orf48, RSL1D1
CD8+ T cells	T.cells.CD8	T.cells.CD8, NA, TMC6, GADD45A, AES, TSC22D3, ZNF609, FLT3LG, LEPROTL1, ABT1, GZMM, APBA2, DNAJB1, C12orf47, PF4, LIME1, PPP1R2, PRF1, CDKN2AIP, PRR5, THUMPD1, RBM3, SFRS7, ZFP36L2, VAMP2, KLF9, TBCC, ZEB1, SF1, ZNF22, ZNF91, HAUS3, MYST3, CAMLG, C19orf6, SLC16A7, CD8A, CD8B
Activated CD8+ T cells	T.cells.CD8.a	T.cells.CD8.a, NA, ADRM1, AHSA1, C1GALT1C1, CCT6B, CD37, CD3D, CD3E, CD3G, CD69, CD8A, CETN3, CSE1L, GEMIN6, GNLY, GPT2, GZMA, GZMH, GZMK, IL2RB, LCK, MPZL1, NKG7, PIK3IP1, PTRH2, TIMM13, ZAP70

Central memory CD8+ T cells	T.cells.CD8.cm	T.cells.CD8.cm, NA, ACTN4, ADAM12, ADCY9, F13A1, FCER1G, FCGR3B, FGF7, FKBP4, GLUD1, GM2A, GUSB, IL1RN, NOL11, NTRK1, RARA, RNF128, SIGLEC1, TNFRSF11A, TOX4, UBA52, ULBP1
Effector memory CD8+ T cells	T.cells.CD8.em	T.cells.CD8.em, NA, ACAP1, APOL3, ARHGAP10, ATP10D, C3AR1, CCR5, CD160, CD55, CFLAR, CMKLR1, DAPP1, FCRL6, FLT3LG, GZMM, HAPLN3, HLA-DMB, HLA-DPA1, HLA-DPB1, IFI16, LIME1, LTK, NFKBIA, SETD7, SIK1, TRIB2
Exhausted CD8+ T cells	T.cells.CD8.exhausted	T.cells.CD8.exhausted, NA, RGS1, HAVCR2, PDCD1, CTLA4, CXCR6, TIGIT, TNFRSF9, PHLDA1, MYO7A, CCL3, ENTPD1, CD27, CD27-AS1, DUSP4, LAYN, HLA-DRA, MYO1E, SRGN, AKAP5, NR4A2, AFAP1L2, TOX, CSF1, COTL1, LYST, SARDH, ETV1, NA, ENTPD1-AS1, IFNG, KIR2DL4, SIRPG, MIR155HG, TBC1D4, ITM2A, CXCL13, TNS3, CLNK, LINC00299, ATP8B4, CREM, SAMSN1, CD38, WHRN, ID3, ICOS, ASB2, TOX2, MIR155, PTGIS, HLA-DMA, RGS2, GAPDH, VCAM1, KLRC4, MS4A6A, APOBEC3G, CD200, PRDM1, SNAP47, IL2RB, WARS, ITGAE, ACP5, ID2, TNFRSF1B, LAG3, SRGAP3, PTPN22, OASL, LAT2, ARID5B, GOLIM4, CLECL1, PDE7B, TNFSF4, FUT8, GLDC, SNX9, NDFIP2, CHST12, CTSD, CCDC141, APOBEC3C, NAB1, HNRNPLL, CD82, FKBP1A-SDCBP2, TNFAIP3, PHEX, GFOD1, PAM, FKBP1A, ZFP36L1, KRT86, ACTN4, CBLB, LSP1, FASLG, TYMP, TTYH3, MIR4632, RNF19A, APOBEC3F, RBPJ, PTMS, HMOX1, BCL2L11, CD7, TRPS1, RAB27A, ARHGAP9, SEMA4A, RIN3, NCF4, TTC24, EPSTI1, TRAF5, ATXN1, FAM3C, INPP5F, SLA, IFI16, CCND2, MTHFD2, SLC2A8, PLPP1, DGKH, ZBED2, NEDD9, PTPN7, IKZF3, CD70, CD63, GPD2, GALM, RALGDS, SEL1L3, P2RY10, CD84, APOBEC3D, IFI27L2, IGFLR1, UBASH3B, TPI1, IFI6, YARS, GGA2, BATF, PDLIM7, ABCG1, PELI1, ATP10D, LINC00963, BTG3, AHI1, UBE2L6, OSBPL3, PON2, KIF20B, BST2, CLSTN3, LIMS1, PTPN11, GCNT1, CAMK1, LY6E, NA, PARP14, PHTF2, GABARAPL1, MTHFD1, ARL3, SSH1, SHFM1, PRKCH, PARK7, LIMK1, JMJD4, PLSCR1, MIR497HG, VAMP5, TFRC, TRAFD1, CD2BP2, CTNNA1, GBP4, PPM1G, IFI35, MAPKAPK3, ZNF79, SPTAN1, EID1, COQ10B, STARD4, CASP3, SKIV2L2, SUSD6, STAM, RHBDD2, PKM, GMDS, STAT3, TBL1XR1, SCO2, PRDX5, DRAP1, GALNT2, CALM3, MIS18BP1, VMP1, PRKAR1A, OAS3, SYNGR2, PSME2, IL21R, MX1, ANXA5, PSTPIP1, ETNK1, MAPRE2, MAD2L2, GTF2I, NFAT5, IRF2, ACSL4, IRF9, GBP1, SLC4A5, TBK1, MAP2K3, ARNT, CTNNB1, RHOH, SAMD9L, FIBP, PFKP, GATA3, GBP2, PMF1, RFX5, NRBP1, TET2, DDX60, SP140, RNF31, PMF1-BGLAP, MCL1, C17orf62, LSM2, GSTO1, AIP, PSMA5, LDHA, EWSR1, OS9, ARPC1B, CAT, ICAM3, ANKRD10, ZMYM5, CAPZA2, NAPA, SNX17, NUDT5, SRI, PPM1M, CTSB, CALCOCO2, BLOC1S2, DCAF11, NASP, CSNK2B, LY6G5B
Granzyme K expressing CD8+ T cells	T.cells.CD8.GZMK	T.cells.CD8.GZMK, NA, GZMK, EML4, PDCD4, DUSP6, PIK3R1
Mucosal-associated invariant CD8+ T cells	T.cells.CD8.MAIT	T.cells.CD8.MAIT, NA, SLC4A10, KLRB1, DPP4, COLQ, ZBTB16, RORC, IL18RAP, CD40LG, IL23R, JAML, ME1, LST1, TMIGD2, NCR3, LTK, CCR6, SESN1, TLE1, IFNGR1, ADAM12, DUSP1, ERN1, TC2N, RUNX2, SLAMF1, IL18R1,

		TSPAN15, CEBPD, GPR171, P2RY14, NR1D1, IL12RB2, FLT4, NRIP1, TBC1D31, SPOCK2, IKZF2, RORA, CD160, DKK3, B3GALT2, HPGD, MBOAT1, CTSH, PHACTR2, SYTL2, CCR1, LOC643733, HACL1, IL4I1, TNF, APOL3, ODF2L, CERK, FKBP11, GPR65, CCR2, TTC39C, GYG1, PBXIP1, TRAT1, PRNP, GALC, MKNK1, TMEM71, NFKBIA, OBFC1, EDEM2, PDK3, PNP
Naïve CD8+ T cells	T.cells.CD8.naive	T.cells.CD8.naive, NA, CCR7, LEF1, SELL, MAL, TCF7, LEF1-AS1, ACTN1, CA6, LDLRAP1, NELL2, TXK, MYC, SERINC5, TRABD2A, NOG, SPINT2, RASGRP2, C1orf162, NT5E, RCAN3, VIPR1, NOSIP, EPHX2, CD248, BACH2, LRRN3, IGF1R, THEM4, TMEM204, CD55, FAM117B, DGKA, PDE3B, TMEM123, ABLIM1, LDHB, STMN3, PRKCQ-AS1, FAM102A, PASK, CAMK4, OXNAD1, TNFSF8, TMEM63A, AIF1, GPR183, C1orf228, RPS8, GLTSCR2, LRRC75A, PIK3IP1, FOXP1, GCSAM, SNED1, APBA2, LMO7, LINC01550, ATM, FKBP5, LRRC75A-AS1, CEP68, RIC3, GPR155, PLEKHB1, ARHGEF18, MAML2, NPM1, GAS5, EIF3L, EIF3E, MLXIP, NDFIP1, RACK1, SH3YL1, TPCN1, PSIP1, C6orf48, ZNF101, SCML4, APEX1, EEF2, R3HDM4, TOMM7, PDK1, TRAP1, NPAT, SMAP2, WDR43, SPTBN1, COQ8A, ARHGAP45, BEX4, UBQLN2, TRMT1, SNURF, FAM60A, SGSM3, ITFG2, EXOSC8, THAP7, GIMAP2
Central memory T cells	T.cells.cm	T.cells.cm, NA, KLF12, CG030, CYLD, TIMM8A, FAM153B, PCNX, CLUAP1, CEP68, DOCK9, POLR2J2, FYB, FOXP1, PDXDC2, HNRNPH1, AQP3, MAP3K1, MLL, ATM, NEFL, NFATC3, PCM1, PSPC1, ATF7IP, CREBZF, ST3GAL1, SNRPN, TXK, PHC3, TRAF3IP3, USP9Y, CASP8, CYorf15B, REPS1, CDC14A, STX16, INPP4B, SLC7A6, NMT2
Effector memory T cells	T.cells.em	T.cells.em, NA, AKT3, DDX17, EWSR1, FLI1, C7orf54, LTK, MEFV, NFATC4, PRKY, TBCD, EZR, GDPD5, TBC1D5
follicular helper T cells	T.cells.fh	T.cells.fh, NA, B3GAT1, CDK5R1, PDCD1, BCL6, CD200, CD83, CD84, FGF2, GPR18, CEBPA, CECR1, CLEC10A, CLEC4A, CSF1R, CTSS, SYNM, DPP4, LRRC32, MC5R, MICA, NCAM1, NCR2, NRP1, PDCD1LG2, PDCD6, PRDX1, RAE1, RAET1E, SIGLEC7, SIGLEC9, TYRO3, CHST12, CLIC3, IVNS1ABP, LGMN
Gamma delta T cells	T.cells.gd	T.cells.gd, NA, ACP5, AQP9, BTN3A2, C1orf54, CARD8, CCL18, CD209, CD33, CD36, CDK5, IL10RB, KLRF1, LGALS1, MAPK7, KLHL7, KRT80, LAMC1, LCORL, LMNB1, MEIS3P1, MPL, FABP1, FABP5, FADD, MFAP3L, MINPP1, RPS24, RPS7, RPS9, DBNL, CCL13
Type 1 T helper (Th1) cells	T.cells.h1	T.cells.h1, NA, CD70, TBX21, ADAM8, AHCYL2, ALCAM, B3GALNT1, BBS12, BST1, CD151, CD47, CD48, CD52, CD53, CD59, CD6, CD68, CD7, CD96, CFHR3, CHRM3, CLEC7A, COL23A1, COL4A4, COL5A3, DAB1, DLEU7, DOC2B, EMP1, F12, FURIN, GAB3, GATM, GFPT2, GPR25, GREM2, HAVCR1, HSD11B1, HUNK, IGF2, RCSD1, RYR1, SAV1, SELE, SELP, SH3KBP1, SIT1, SLC35B3, SIGLEC10, SKAP1, THUMPD2, TIGIT, ZEB2, ENC1, FAM134B, FBXO30, FCGR2C, STAC, LTC4S, MAN1B1, MDH1, MMD, RGS16, IL12A, P2RX5, CD97, ITGB4, ICAM3, METRNL, TNFRSF1A, IRF1, HTR2B, CALD1, MOCOS, TRAF3IP2, TLR8, TRAF1, DUSP14
Type 17 T helper (Th17) cells	T.cells.h17	T.cells.h17, NA, IL17A, IL17RA, C2CD4A, C2CD4B, CA2, CCDC65, CEACAM3, IL17C, IL17F, IL17RC, IL17RE, IL23A, ILDR1, LONRF3, SH2D6, TNIP2, ABCA1, ABCB1,

		ADAMTS12, ANK1, ANKRD22, B3GALT2, CAMTA1, CCR9, CD40, GPR44, IFT80
Type 2 T helper (Th2) cells	T.cells.h2	T.cells.h2, NA, ASB2, CSRP2, DAPK1, DLC1, DNAJC12, DUSP6, GNAI1, LAMP3, NRP2, OSBPL1A, PDE4B, PHLDA1, PLA2G4A, RAB27B, RBMS3, RNF125, TMPRSS3, GATA3, BIRC5, CDC25C, CDC7, CENPF, CXCR6, DHFR, EVI5, GSTA4, HELLS, IL26, LAIR2
Regulatory T Cells	T.cells.reg	T.cells.reg, NA, FOXP3, LAYN, IKZF2, TNFRSF18, TIGIT, TNFRSF9, CTLA4, RGS1, CCR8, IL2RA, CXCR6, DUSP4, TNFRSF4, CTSC, CD27, PHLDA1, CD27-AS1, TBC1D4, LAIR2, RTKN2, SDC4, ENTPD1, NAMPT, IL2RB, CREM, IL32, TNFRSF1B, BATF, STAM, SH2D2A, MIR4435-2HG, CLEC7A, ICOS, F5, IL1R1, MIR4632, CD74, GAPDH, DNPH1, RHOC, VDR, PTTG1, IKZF4, TNFAIP3, NEDD9, GBP5, TYMP, IL12RB2, SAMSN1, CARD16, ACP5, HAVCR2, ENTPD1-AS1, TNFRSF13B, SPATS2L, ASB2, IL1R2, SERPINE2, LAPTM4B, GADD45A, PHTF2, FANK1, SIRPG, PMAIP1, CXCR3, NDFIP2, CD7, SAT1, GK, PRDM1, CCDC22, PELI1, CCDC141, MAF, ETV7, WHRN, BTG3, PIM2, MZB1, GBP2, C15orf53, COL9A2, BEX3, ICA1, TNIP3, LYST, CYTOR, CSF1, CTTNBP2NL, REREP3, METTL7A, VAV3, CLNK, SYNGR2, ARHGEF12, IL21R, HLA-DQA1, GPX1, ATP1B1, TOX, HNRNPLL, CORO1B, GADD45G, TFRC, SNX9, SUOX, SOX4, HLA-DMA, DDIT4, LGALS3, TOX2, EBI3, TBC1D8, CREB3L2, ZFP36L1, MAST4, LTA, CASP1, SLAMF1, CDKN2A, SCO2, ZBED2, PDE4A, KAT2B, TTN, CDKN1A, MICAL2, CD58, NCF4, CD82, CD83, PTPRJ, ARID5B, CCR1, DUSP16, DPYSL2, IFI6, GLRX, TSPAN13, CSF2RB, TNS3, CCR4, CD80, C3AR1, ADAT2, PKM, IL21R-AS1, MYO1E, CTSD, CD79B, RBPJ, TNFRSF8, GCNT1, LMCD1, CADM1, HACD1, EPSTI1, CCNG2, CCND2, ERI1, ZC2HC1A, OAS1, AKAP5, BIRC3, ZBTB32, RAB11FIP1, CD177, INPP5F, TPP1, HSPA1B, FKBP1A-SDCBP2, TRPS1, FKBP1A, TTYH3, SLC41A1, SLA, MYO5C, KCNN4, PHACTR2, CCDC50, RHBDD2, BST2, ADPRH, CDIP1, IGFLR1, RPS27L, HSPA1A, TRAF1, SLC35F2, LINC00963, SPOCK2, SURF4, MAP1LC3A, GALM, ENO1, CTNNA1, CARD17, NAB1, UGP2, UCP2, EDARADD, BRE-AS1, GABARAPL1, FAM53B, HPGD, MIR497HG, CEACAM1, GRINA, CALM3, PHPT1, OTUD5, FANCL, PTP4A3, CD70, GOLGA8B, NCOA3, LOC389831, MAGEH1, PRNP, TET2, APLP2, DUSP10, MAN1A2, IRF5, FAS, MAP2K3, LRRC61, FAM110A, TULP4, LAT2, CTNNB1, PRKAR1A, SSH1, STAMBPL1, LASP1, NPTN, ISG15, TRAF3, TMEM154, ATP6V0A1, PARK7, PDIA6, GRN, HSPB1, ACSL4, PPM1G, SMC4, CD59, GOLGA8A, ZC3H12D, VCP, FYCO1, MYO5A, CTSA, PSMB3, COX5A, ARPP19, PPP1CB, SLC16A1, LDLRAD4, UXS1, ABI2, LAP3, PLTP, FBLN7, ANKRD10, SERPINB9, DYNLL1, ATP6V1C2, MRPS6, GTF3C6, NFAT5, TPI1, GPI, PRDX1, GBP4, RFX5, NINJ2, INPP1, IL12RB1, ARPC1B, TNIP1, WSB1, FMNL3, TP53INP1, VMP1, KDM5B, PIM3, ATOX1, ACTA2, ASXL2, ADCY3, SMPD3, CKAP2, PGK1, IRF4, AKIRIN2, GRAMD3, AKIP1, POU2F2, AFTPH, ACOT9, C16orf87, NUDT5, ZNF280C, KIF20B, SMS, WDR83OS, METTL8, PAK2, WDFY1, GSTO1, TLK1, FOXO1, SGPP1, RNF187, FAM104A, BRE, SLC5A3, NDUFA13, ACTR3C, PSMB6, YJEFN3, RCBTB1, HPSE, SEC61A2,

	P2RY10, THADA, DDX24, PRDX3, ARPC1A, HERPUD1, MBOAT7, C21orf91, SLC12A6, TSPYL2, RAP1A, MAPKAPK3, ATP13A3, TRIM69, SQSTM1, UBE2L6, GABPB1, SH3BGRL, C9orf16, MCL1, BACH1, SFT2D1, PCK2, PLEKHG2, HTATIP2, PRDX5, GABARAP, MCM5, ALDH3A2, CMTM6, SGMS1, ISOC1, MCCC2, GATA3, LMBRD1, ADAM10, ADORA2A, LRPAP1, SEC14L1, KIF5B, COMMD3, SDF4, OAS3, SRA1, MGAT1, LEPROT, SLC3A2, PAIP2, LY75, DYNLRB1, NINJ1, HDAC7, ZNF292, ARHGAP9, NPC2, TPMT, IFNAR2, VEZT, SGSH, DYNC1I2, NSF, OS9, IL10RB, HPRT1, C5orf45, PSMC2, TMX1, VPS54, STARD7, NME3, NDUFC2-KCTD14, SUMO1, PSMB1, TM9SF2, HADHB, ADI1, SLC25A38, DDB2, NDUFC2, ICAM3, LAPTM4A, LY75-CD302, PSMA2, OCIAD2, ZC3H7A, ECH1, CLIP1, HSD17B10, C7orf25, NAPA, PSMD11, CBX5, CACYBP, TOM1, HADHA, GOT2, MX1, UBE2N, COG1, MIIP, COPZ1, PSEN1, NBR1, USP48, EIF4H, IRF9

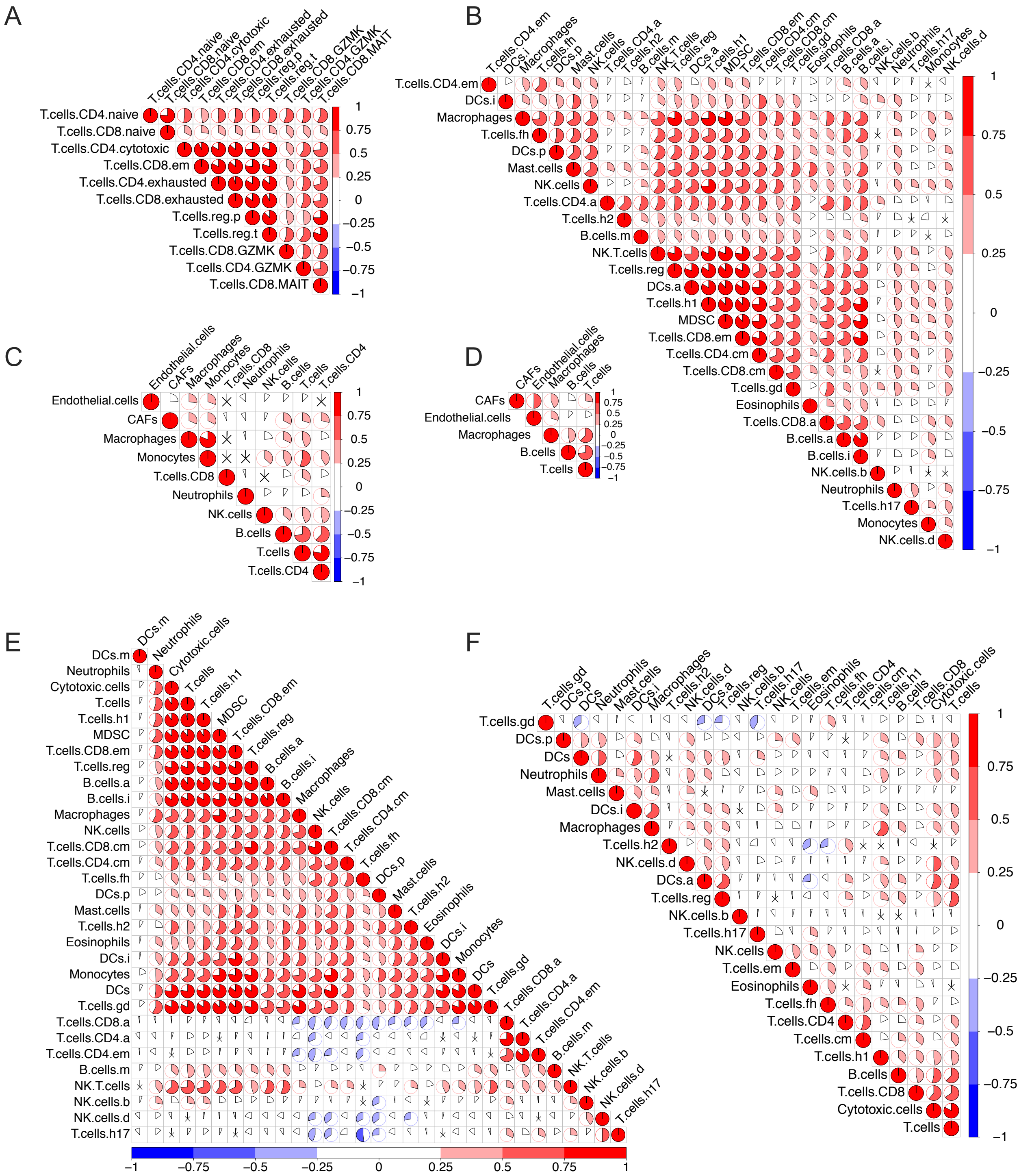

Figure S1. Pearson's correlation coefficient methods identifies the correlation of the ssGSEA scores across the gene sets provided by 6 published study. The ssGSEA scores for most immune cell populations obtained using the gene sets from Zheng et al. [1] (A), Charoentong et al. [2] (B), Racle et al. [3] (C), and Tirosh et al. [4] (D), exhibit a positive high or mild correlation. The ssGSEA scores for most immune cell populations obtained using the gene sets from Angelova et al. [5] (E), are either highly correlated or mildly anti-correlated. The ssGSEA scores for a few immune cell populations obtained using the gene sets from Bindea et al. [6] (F) are mid anti-correlated.

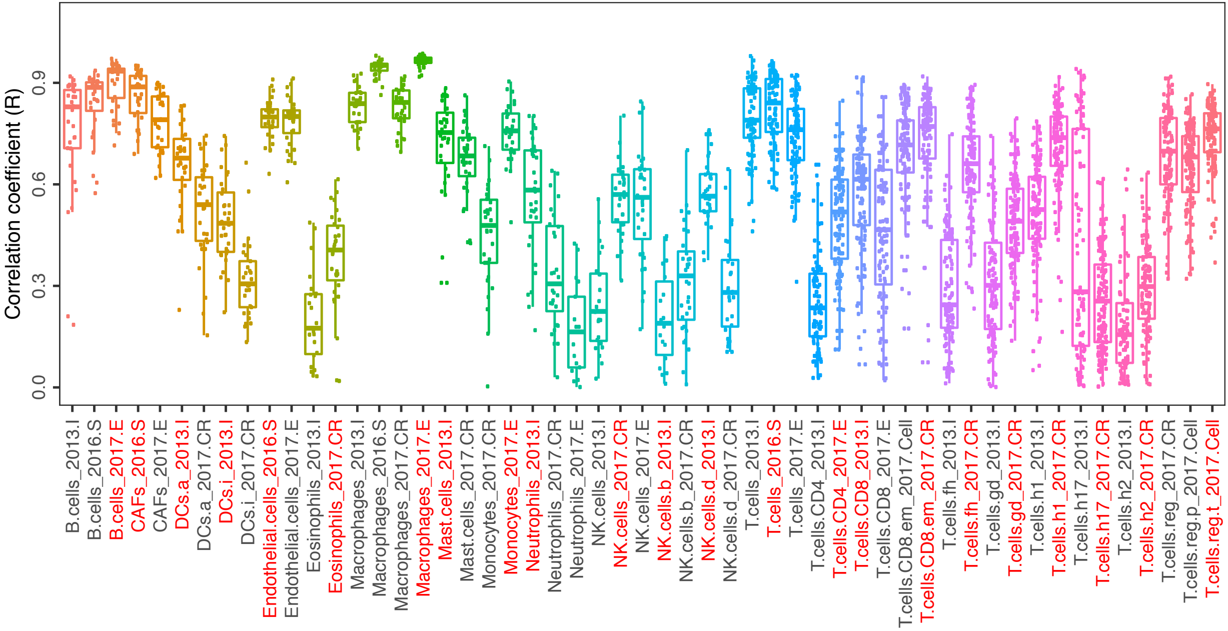

Figure S2. Agreement between ssGSEA scores and known markers of immune cell populations.

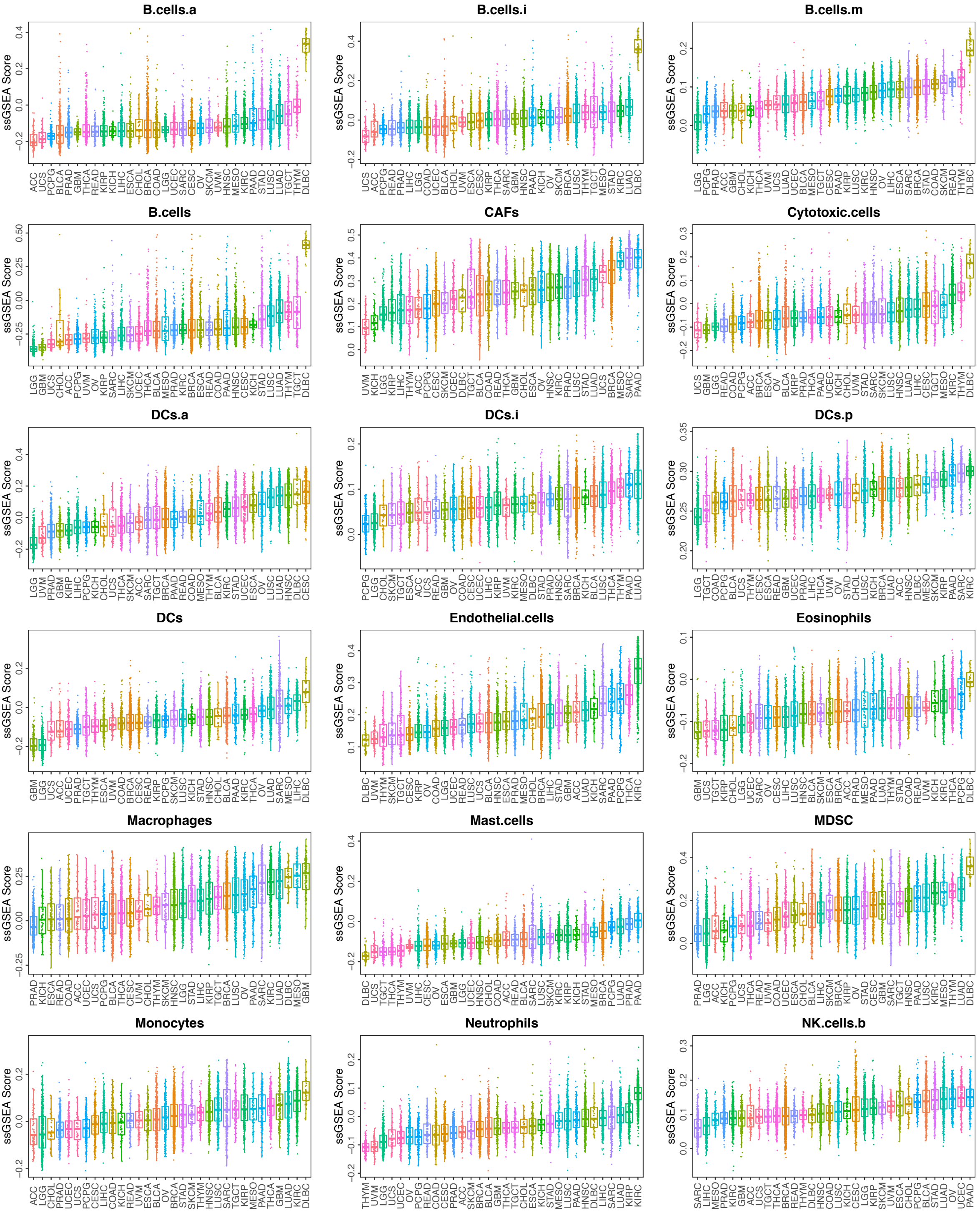

Figure S3. Distribution of the median ssGSEA scores of 46 immune cell populations across the tumors of each cancer type.

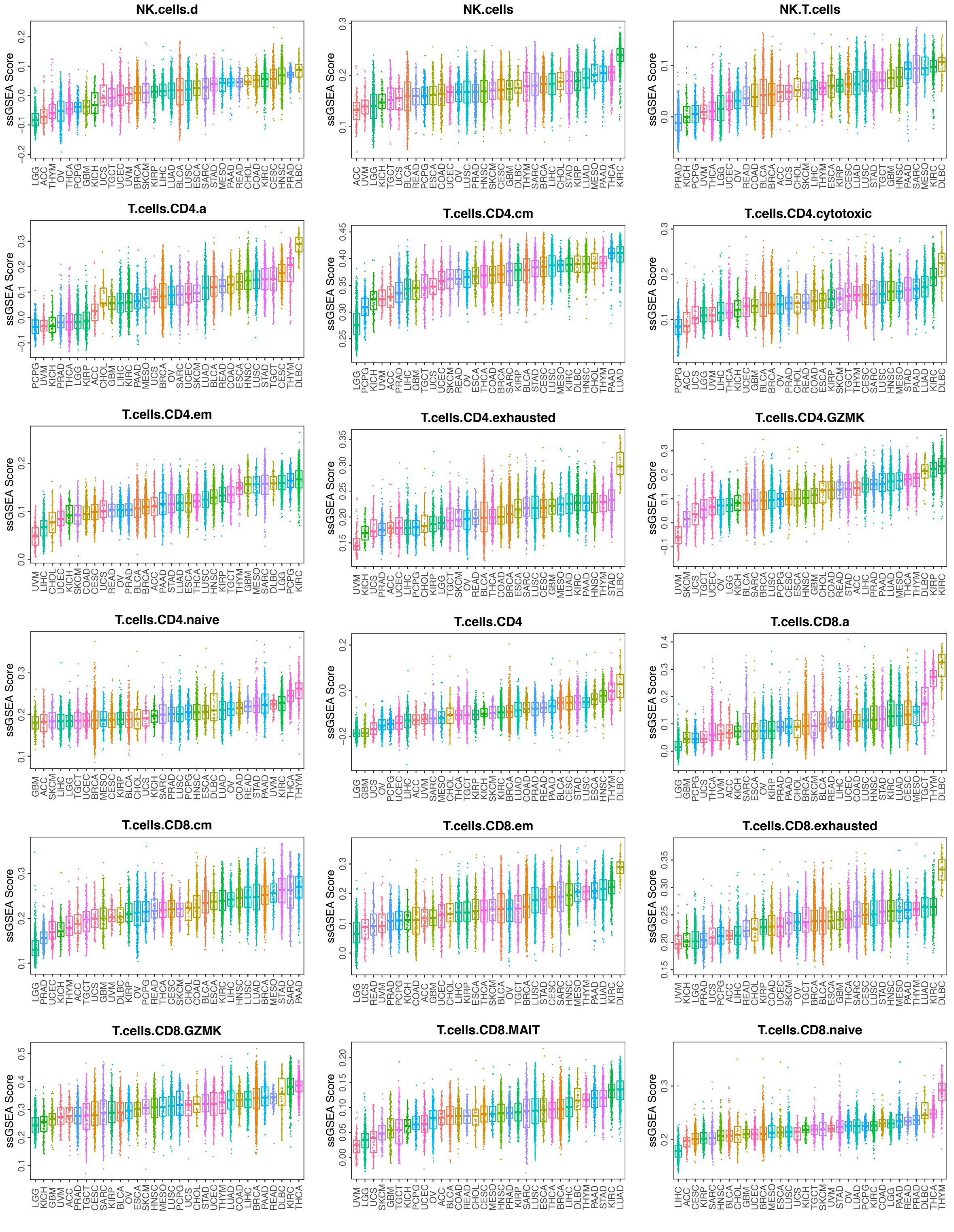

Figure S3 Continued. Distribution of the median ssGSEA scores of 46 immune cell populations across the tumors of each cancer type.

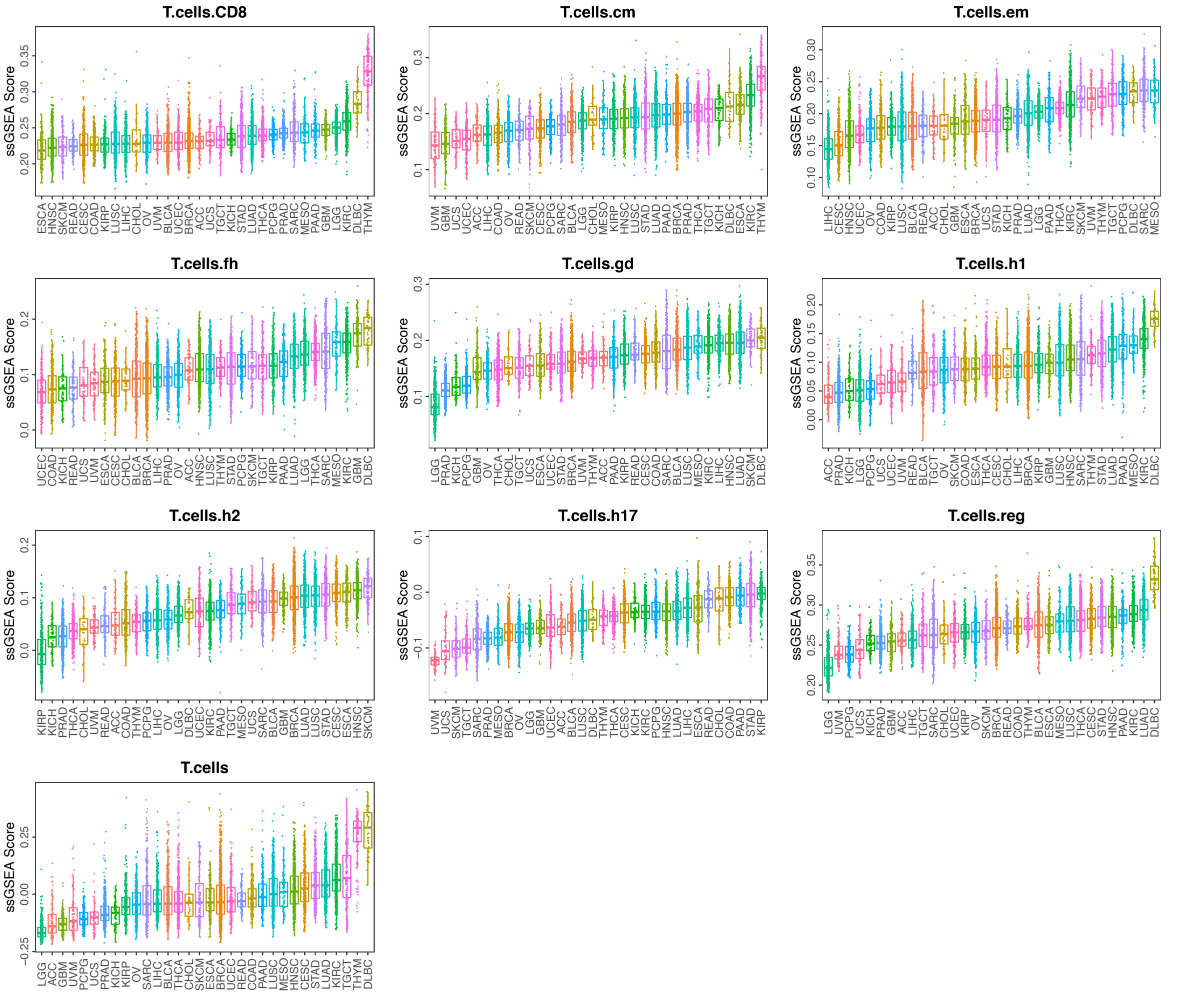

Figure S3 Continued. Distribution of the median ssGSEA scores of 46 immune cell populations across the tumors of each cancer type.

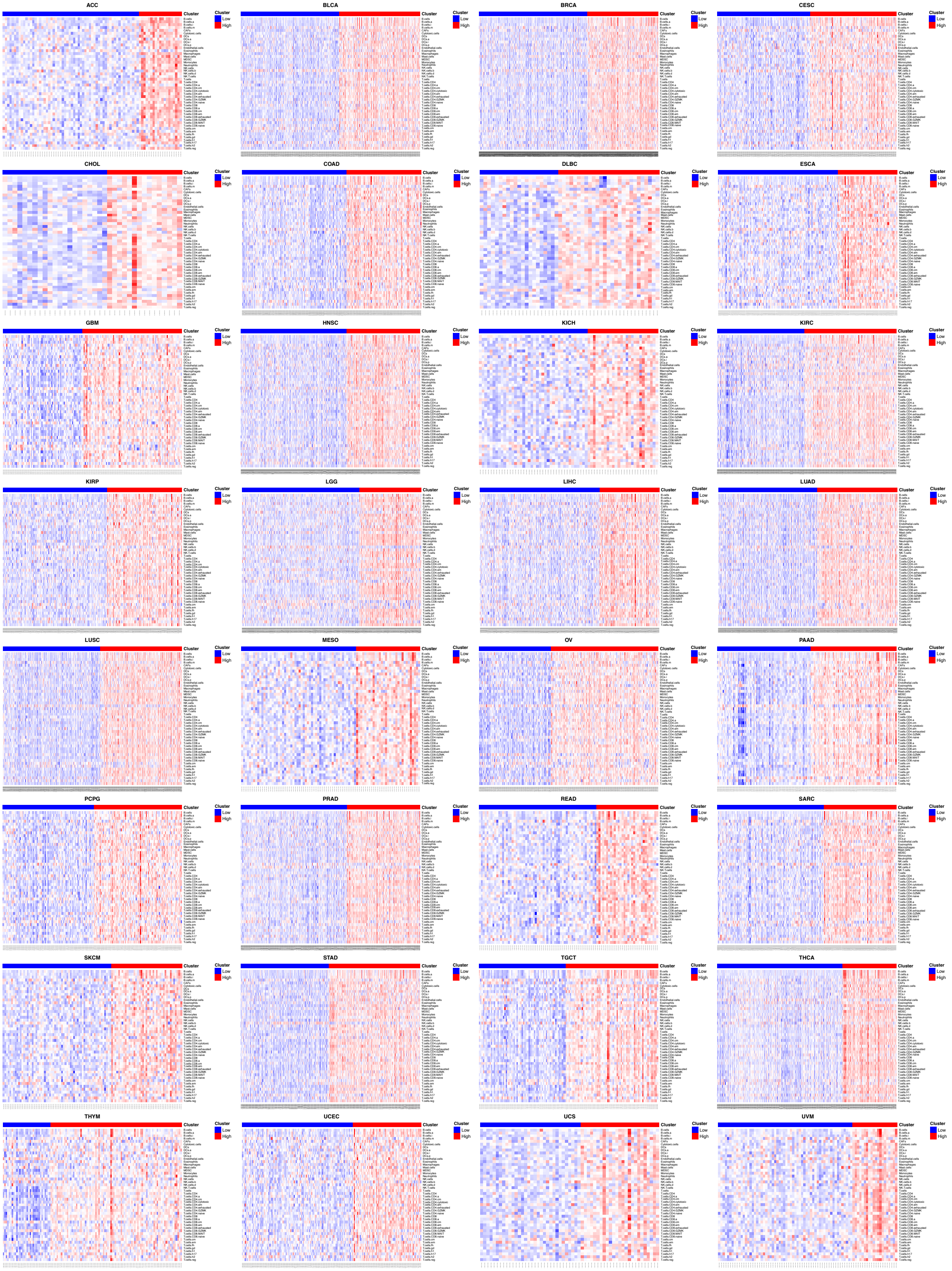

Figure S4. Unsupervised clustering using the ssGSEA scores from 46 immune cell populations on each cancer type.

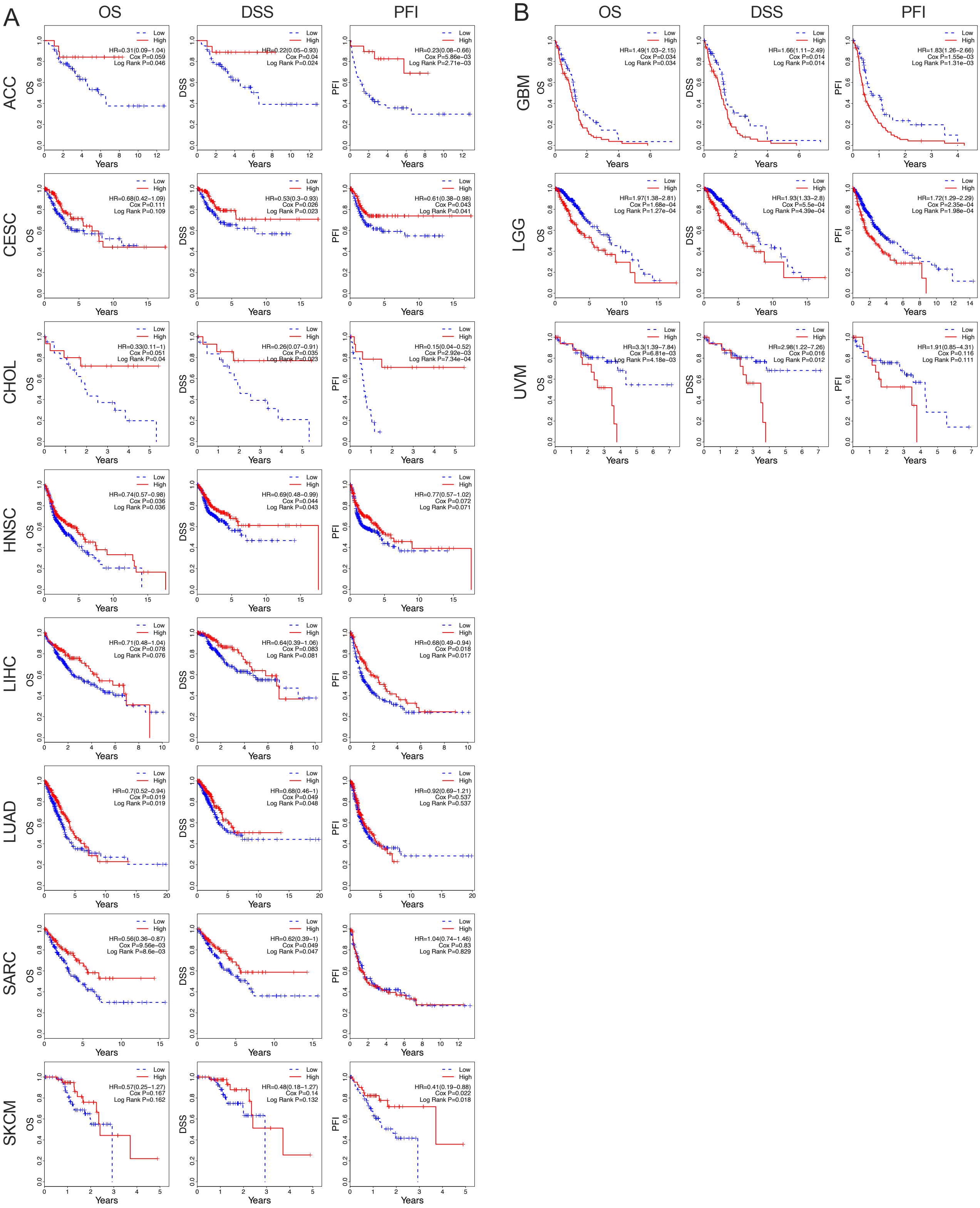

Figure S5. Kaplan-Meier survival curves identify the survival differences between two immunophenotypes across 11 cancer types. A: High immune cell infiltration shows a better prognosis in ACC, CESC, CHOL, HNSC, LIHC, LUAD, SARC, and SKCM. B: High immune cell infiltration shows a worse prognosis in GBM, LGG, and UVM.

References

1. Zheng, C., et al., Landscape of Infiltrating T Cells in Liver Cancer Revealed by Single-Cell Sequencing. Cell, 2017. 169(7): p. 1342-1356 e16.
2. Charoentong, P., et al., Pan-cancer Immunogenomic Analyses Reveal Genotype-Immunophenotype Relationships and Predictors of Response to Checkpoint Blockade. Cell Rep, 2017. 18(1): p. 248-262.
3. Racle, J., et al., Simultaneous enumeration of cancer and immune cell types from bulk tumor gene expression data. Elife, 2017. 6.
4. Tirosh, I., et al., Dissecting the multicellular ecosystem of metastatic melanoma by single-cell RNA-seq. Science, 2016. 352(6282): p. 189-96.
5. Angelova, M., et al., Characterization of the immunophenotypes and antigenomes of colorectal cancers reveals distinct tumor escape mechanisms and novel targets for immunotherapy. Genome Biol, 2015. 16: p. 64.
6. Bindea, G., et al., Spatiotemporal dynamics of intratumoral immune cells reveal the immune landscape in human cancer. Immunity, 2013. 39(4): p. 782-95.
